# *Kif3a* Controls Murine Nephron Number Via GLI3 Repressor, Cell Survival, and Gene Expression in a Lineage-Specific Manner

**DOI:** 10.1371/journal.pone.0065448

**Published:** 2013-06-07

**Authors:** Lijun Chi, Alevtina Galtseva, Lin Chen, Rong Mo, Chi-chung Hui, Norman D. Rosenblum

**Affiliations:** 1 Program in Developmental and Stem Cell Biology, The Hospital for Sick Children, University of Toronto, Toronto, Ontario, Canada; 2 Division of Nephrology, The Hospital for Sick Children, Toronto, Ontario, Canada; 3 Department of Molecular Genetics, University of Toronto, Toronto, Ontario, Canada; 4 Department of Paediatrics, University of Toronto, Toronto, Ontario, Canada; 5 Department of Laboratory Medicine and Pathobiology, University of Toronto, Toronto, Ontario, Canada; 6 Department of Physiology, University of Toronto, Toronto, Ontario, Canada; The University of Manchester, United Kingdom

## Abstract

The primary cilium is required during early embryo patterning, epithelial tubulogenesis, and growth factor-dependent signal transduction. The requirement for primary cilia during renal epithelial-mesenchymal tissue interactions that give rise to nephrons is undefined. Here, we used *Cre-*mediated recombination to generate mice with *Kif3a* deficiency targeted to the ureteric and/or metanephric mesenchyme cell lineages in the embryonic kidney. Gradual loss of primary cilia in either lineage leads to a phenotype of reduced nephron number. Remarkably, in addition to cyst formation, loss of primary cilia in the ureteric epithelial cell leads to decreased expression of *Wnt11* and *Ret* and reduced ureteric branching. Constitutive expression of GLI3 repressor (*Gli3^Δ699/+^*) rescues these abnormalities. In embryonic metanephric mesenchyme cells, *Kif3a* deficiency limits survival of nephrogenic progenitor cells and expression of genes required for nephron formation. Together, our data demonstrate that *Kif3a* controls nephron number via distinct cell lineage-specific mechanisms.

## Introduction

Primary cilia are microtubule-based organelles that function as signaling centers during development and cell differentiation [1]. The primary cilium arises in a quiescent cell from the basal body as a microtubule-based plasma membrane-invested cytoskeletal structure termed the axoneme. Cilia assembly and maintenance and growth of the axoneme is mediated by a kinesin motor protein-based transport process termed intraflagellar transport (IFT), by which particles are transported in a bidirectional manner along the axoneme [2]. Deficiency of KIF3A, a component of the kinesin II motor complex, disables anterograde IFT, and causes both failure of formation and maintenance of the primary cilium [3]. A critical role for the primary cilium during embryogenesis was initially demonstrated by the finding that mice with *Kif3a* deficiency lack nodal cilia and exhibit defects in left-right asymmetry [3]. Many human congenital malformation syndromes are caused by mutations in proteins that are localized to cilia and ciliary basal bodies [1]. Some of the mutated proteins are downstream effectors of the Hedgehog (Hh), WNT and FGF signaling pathways. Hh ligands signal by binding the cell surface protein Patched (PTC), which functions as a constitutive inhibitor of Smoothened (SMO). In the absence of Hh ligand, inactive SMO promotes the processing of full length GLI3 to a C-terminally truncated transcriptional repressor, GLI3 repressor (GLI3R). Hh activates SMO, leading to the blockage of GLI3 processing and the nuclear translocation of full-length GLI proteins to induce transcription. Several lines of evidence implicate the primary cilium in mammalian Hh signaling. First, disruption of Hh signaling generates a phenotype very similar to that described in embryos with deficiency of IFT proteins [4]. Second, PTC, SMO, and GLI are localized to the primary cilium [5]–[7]. Third, IFT proteins act downstream of PTC1 and SMO and upstream of GLI proteins [4], [8]. Cilia defects alter the ratio of GLI activator to GLI3R resulting in aberrant Hh signaling [1]. The primary cilium is also implicated in WNT signaling since NPHP2 (inversin), NPHP3, and GLIS2, each of which promotes noncanonical WNT signaling, are localized to the cilium. Inactivation of any of these noncanonical WNT effectors increases canonical WNT activity [9], [10]. In contrast to Hh and WNT signaling, the role of the primary cilium in regulating FGF signaling is largely unknown. FGFs have been shown to regulate cilia length [11] but a role for the cilium in regulating FGF signaling has not been demonstrated previously. However, the localization of FGF receptors to cilia in murine airway cells suggests a possible role for the cilium in regulating FGF signaling [12].

The discovery that proteins mutated in polycystic kidney diseases are localized to the primary cilium identified the primary cilium as critical to renal epithelial cell differentiation [13]. In direct support of this cilia-dependent function, kidney-specific inactivation of *Kif3a* in the ureteric epithelial cell lineage inhibits ciliogenesis and induces epithelial cysts [14]. The observation that nephron formation may be impaired in mice with deficiency of NPHP2 (inversin), a cilia-localized protein and polycystic kidney disease gene [9] suggests that primary cilia may function during stages of renal development that control nephron formation and which precede epithelial differentiation.

Nephron formation is dependent on inductive mesenchymal-epithelial tissue interactions between the ureteric bud (an epithelial tubule) and the metanephric blastema (a mesenchymal tissue). Metanephric mesenchyme cells adjacent to ureteric branch tips are induced to form nephrogenic precursors that constitute the mature nephron (glomerulus, proximal tubule, loop of Henle, and distal tubule), a process that has been termed nephrogenesis. In turn, the ureteric bud and its branches are stimulated to undergo successive branching events in response to signals by adjacent mesenchyme cells, resulting in formation of the collecting ducts, calyces and pelvis, a process termed renal branching morphogenesis [15]. Investigation of the molecular mechanisms that control nephrogenesis and branching morphogenesis has elucidated critical roles for signaling by Hh, WNT and FGF proteins. Sonic Hh (Shh) controls inductive tissue interactions during murine kidney development by inhibiting formation of GLI3 repressor [16]. During branching morphogenesis, GLI3R plays a critical role in distal ureteric branch tips by promoting the expression of *Ret* and *Wnt11*, both of which are required for ureteric branching [17]. Canonical WNT signaling is required for renal branching morphogenesis [18] and formation of nephrogenic precursors in response to WNT9b and WNT4 [19]–[21]. Nephron formation is also dependent on expression of *FGF8* by metanephric mesenchyme cells. Deficiency of *Fgf8* abrogates expression of *Wnt4* and limits nephron formation to stages prior to the formation of the glomerulus [22].

Here, we tested our hypothesis that the primary cilium is required during growth factor-mediated renal mesenchymal-epithelial interactions. We investigated our hypothesis by generating mouse strains with deficiency in *Kif3a* in all kidney cells or in the ureteric or metanephric mesenchyme cell lineage. Our results demonstrate that *Kif3a* deficiency and subsequent loss of primary cilia is accompanied by a decrease in the number of nephrons. Analysis of mice with lineage-specific *Kif3a* deficiency showed that *Kif3a* performs distinct functions in ureteric and metanephric mesenchyme cells. In ureteric cells, *Kif3a* deficiency disrupts ureteric branching and expression of *Ret* and *Wnt11,* which act in concert to promote ureteric branching. Remarkably, constitutive expression of GLI3R in *Kif3a*-deficient ureteric cells rescues each of these abnormalities. Analysis of mice with *Kif3a* deficiency in metanephric mesenchyme cells revealed two further mechanisms by which *Kif3a* controls nephron number. First, *Kif3a-*deficient cells exhibit reduced survival, negatively impacting the mass of mesenchyme cells that can contribute to nephrons. Second, expression of FGF8 and its downstream effectors by *Kif3a-*deficient cells is markedly reduced. Yet, expression of Hh signaling effectors is unaffected. Together, these results demonstrate a fundamental role for *Kif3a* and the primary cilium in controlling nephron number during murine kidney development.

## Results

### *Kif3a* Deficiency Decreases Nephron Formation in the Murine Kidney

We initiated investigation of primary cilium function during renal morphogenesis by examining the cellular distribution of primary cilia in distinct lineages that give rise to the kidney. Acetylated alpha-tubulin (α-AcT) is expressed in the ciliary axoneme and the basal body from which the axoneme emerges; expression on the apical cell surface marks the primary cilium. Examination of α-AcT expression at E11.5, the stage at which the ureteric bud invades the metanephric blastema, demonstrated expression in virtually all ureteric and mesenchyme cells (Figure S1A’ and S1A’’). By E13.5 and E15.5, α-AcT could be clearly localized to the apical surface of ureteric cells (Figure S1B’, S1B’’, S1C’, and S1C”), as well as the mesenchyme-derived structures, condensing mesenchyme, vesicles and S-shaped bodies (Figure S1D’ and S1D”), that precede formation of the mature nephron. Together, these data indicate that primary cilia are formed during early stages of ureteric branching and nephron formation.

**Figure 1:**
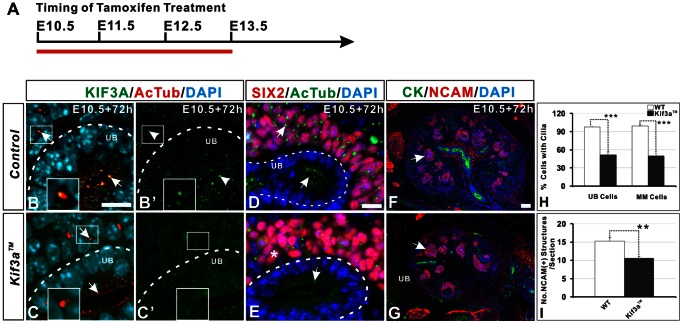
Loss of primary cilia and decreased nephron number in mice with Tamoxifen-induced *Kif3a* deficiency. (A) Chart showing embryonic stage at which Tamoxifen was injected (E10.5) and at which kidneys were retrieved (E13.5) for analysis (red line). (B, B’) KIF3A co-localizes with α-AcT in both ureteric bud and metanephric mesenchyme cells in WT kidney at E13.5. Insert box shows high-resolution image of KIF3A located in a primary cilium in a metanephric mesenchyme cell. (C, C’) Expression of *Kif3a* is largely undetectable 72 hours after Tamoxifen administration to pregnant *Kif3a^loxP/loxP^* mice which had been crossed to *Cre-ER™;Kif3a^+/−^* mice. (D, E) The number of primary cilia (green) is markedly decreased in both SIX2-positive cells (nephrogenic precursors) and in ureteric cells *Kif3a™* kidney (E, asterisk) compared to WT kidney (D). (F, G) Imaging of NCAM-positive nephrogenic precursor structures (arrows). The number of precursors is decreased in *Kif3a™* mice (G) compared to WT mice (F). (H) Quantitation of the number of cells with a primary cilium. Tamoxifen administration to *Kif3a™* embryos decreases cilia number by approximately 50% in both ureteric and metanephric mesenchyme cells (***, P<0.001). (I) Quantitation of the number of NCAM-positive nephrogenic precursor structures in tissue sections reveals a significant decrease in *Kif3a™* mice compared with WT mice (**, P<0.01). WT, wild type; Scale bar: 25 micrometer.

We investigated the functional contribution of the primary cilium to renal development by generating mice with loss of primary cilia in both ureteric and metanephric mesenchyme cells. KIF3A is a component of the microtubule heterotrimeric kinesin II motor complex, which mediates anterograde IFT. *Kif3a* deficiency disables anterograde IFT and leads to failure in formation and maintenance of cilia [3]. Since germline deficiency in *Kif3a* is embryonic lethal prior to the onset of kidney development [3], we used a conditional *Kif3a^loxP^* allele [3] and a Tamoxifen-inducible *Cre* mouse strain [23] to generate *Cre-ER™;Kif3a^loxp/−^* (termed *Kif3a™* ) mice. Administration of Tamoxifen (3mg/40g body weight) prior to E10.5 induced embryonic demise prior to kidney formation. In contrast, embryos of pregnant dams treated with Tamoxifen at E10.5 survived until shortly after E13.5 (Figure 1A), thus providing a means to analyze primary cilium function.

Loss of KIF3A in Tamoxifen-treated mice was confirmed using anti-KIF3A antibody (Figure 1B, 1C’). In control mice (WT mice injected with Tamoxifen at E10.5), KIF3A and α-AcT co-localized in ureteric and metanephric mesenchyme cells (Figure 1B, B’). In ureteric branches, characterized by a lumen, co-localization of KIF3A and α-AcT was restricted to the apical cell surface (Figure 1B, arrow located in UB apical domain). In contrast, expression of KIF3A was markedly diminished in *Kif3a™* mice (Figure 1C’). Further, co-localization of KIF3A with α-AT in the apical domain of epithelial cells was not detected (Figure 1C). We assessed the impact of KIF3A deficiency on primary cilia by counting the fraction of ureteric cells and nephrogenic metanephric mesenchyme (SIX2-positive) cells with primary cilia by imaging these respective cells in three randomly selected optical fields in a 5 micrometer sagittal tissue section generated from the mid-point of each kidney (n = 6 mice/group) (Figure 1D and 1E). The number of cells with primary cilia was decreased by 54% in each of these cell populations in *Kif3a*-deficient mice compared with controls (Figure 1H). Next, we determined the effect of KIF3A and primary cilium loss on kidney development. Ureteric branches and nephrogenic precursors were identified in tissue sections with cytokeratin (CK) and NCAM, respectively (Figure 1F and 1G). Both ureteric branches and nephron precursors formed normally in mutant mice. The number of NCAM-positive nephrogenic precursors was determined for each kidney in five tissue sections - a mid-sagittal section and two sections generated 40 micrometer and 80 micrometer in both directions from the mid-sagittal section, resulting in a total of five sections. Quantitation of NCAM-positive nephrogenic precursors demonstrated a 34% decrease in mutant mice (no. NCAM-positive structures/section, WT versus *Kif3a™*: 15.2±2.96 versus 10±1.5, p = 0.001, n = 4 mice/group) (Figure 1I). These data demonstrate that *Kif3a* controls the number of nephrons formed during renal embryogenesis.

### *Kif3a* Functions in a Cell-lineage Specific Manner to Control Nephron Number

**Figure 2:**
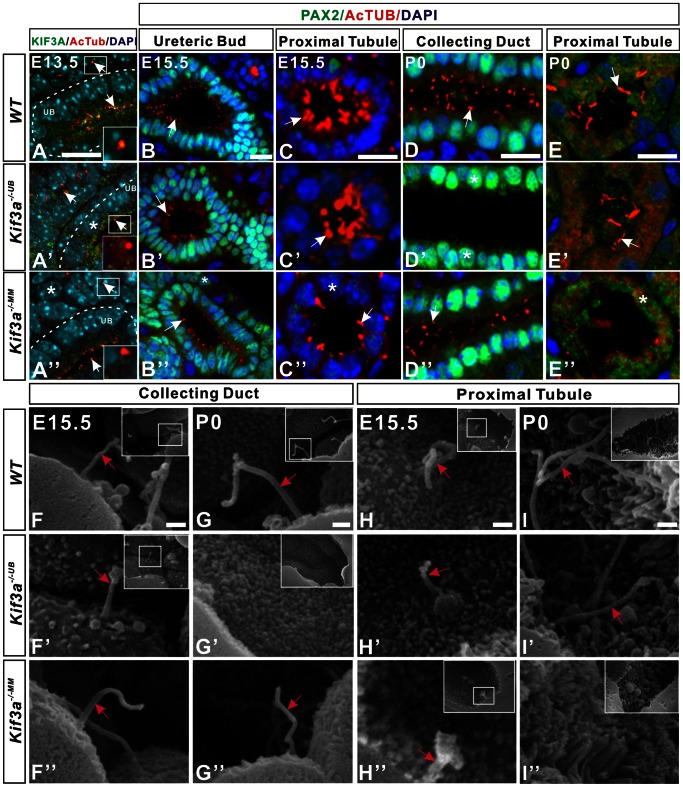
Gradual loss of primary cilia after induction of *Kif3a* deficiency in ureteric or metanephric mesenchyme cell lineages. (A, A’, A’’) KIF3A expression in E13.5 kidney tissue. (A) KIF3A co-localizes with α-AcT (arrow) on the apical surface of ureteric cells and in metanephric mesenchyme cells (box) in WT mice. (A’) In *Kif3a^−/−UB^* mice, KIF3A expression (arrow) is markedly decreased in ureteric cells (box) but is comparable to WT in metanephric mesenchyme cells. (A”) In *Kif3a^−/−MM^* mice, KIF3A expression (arrow) is lost in metanephric mesenchyme cells (box) but is retained in ureteric cells. (B, B’, B’’) Primary cilia (arrow) in ureteric cells at E15.5. The number of cilia is decreased in *Kif3a^−/−UB^* mice (B’) but is comparable to WT (B) in *Kif3a^−/−MM^* mice (B”). (C, C’, C’’) Primary cilia (arrow) in proximal tubules at E15.5. The number of cilia is decreased in *Kif3a^−/−MM^* mice (C”) but is unaffected in *Kif3a^−/−UB^* mice (C’) compared to WT (C). (D, D’, D”) Primary cilia in collecting ducts at P0. Cilia (red) are absent from the collecting duct lumen and the cell body (asterisk) (D’) but are unaffected in *Kif3a^−/−MM^* mice. (E, E’, E’’) Primary cilia (arrow) in proximal tubules at P0. Cilia (arrows) are absent from the tubule lumen in *Kif3a^−/−MM^* mice (E’’). Expression of α-AcT (asterick) is visible within the body of some proximal tubule cells. Cilia are unaffected in the proximal tubule of *Kif3a^−/−UB^* mice (E’). (F–G”) SEM of collecting ducts in WT (F, G), *Kif3a^−/−UB^* (F’, G’), and *Kif3a^−/−MM^* (F”, G”) mice. In *Kif3a^−/−UB^* mice, cilia (arrow) are decreased in number at E15.5 (F’, box) and are absent at P0 (G’, box) compared to WT (F, G, boxes). Cilia are unaffected in *Kif3a^−/−MM^* mice (F”, G”). (H–I”) SEM of proximal tubules in WT (H, I), *Kif3a^−/−UB^* (H’, I’), and *Kif3a^−/−MM^* (H”, I”) mice. In *Kif3a^−/−MM^* mice, cilia (arrow) are shorter at E15.5 (H”, box) and are absent at P0 (I”, box) but are unaffected in *Kif3a^−/−UB^* mice (H’, I’) versus WT controls (H, I, boxes). MM, metanephric mesenchyme; UB, ureteric bud; WT, wild type. Scale bars: A–E’’, 25 micrometer, F–I’’, 5 micrometer.

The short-term viability of embryos in Tamoxifen-treated pregnant mice limited the availability to investigate mechanisms underlying the requirement for *Kif3a* during nephron formation. To address this limitation, we generated mice with loss of *Kif3a* targeted specifically to the ureteric (*Kif3a^−/−UB^* mice) or metanephric mesenchyme (*Kif3a^−/−MM^* mice) lineages, using *Hoxb7-CreEGFP* [24] and *Rarb2-Cre* [25] mouse strains, respectively. *Kif3a^−/−UB^* and *Kif3a^−/−MM^* mouse embryos were generated in the proportion predicted by Mendelian segregation and survived to birth. The efficiency and specificity of *Kif3a* deletion was demonstrated by analyzing *Kif3a* mRNA expression in ureteric bud and metanephric mesenchyme tissue fractions isolated at E11.5 using quantitative PCR. *Kif3a* mRNA was reduced by over 95% in ureteric and metanephric mesenchyme cells in *Kif3a^−/−UB^* and *Kif3a^−/−MM^* mice, respectively, but was not significantly decreased in cells that were not targeted by the respective *Cre* alleles (Figure S2). KIF3A protein expression was examined by immunofluorescence in embryonic kidney tissue. Comparison of anti-KIF3A antibody-generated signals on the apical surface of control and mutant cells revealed specific identification of KIF3A. In control embryos, KIF3A (green color) co-localized with α-AcT (red color) on the apical surface of ureteric cells (Figure 2A, UB: arrow) and in mesenchyme cells (Figure 2A, arrow in box). In *Kif3a^−/−UB^* mice, KIF3A was lost in ureteric cells (Figure 2A’, box inset) but was expressed in metanephric mesenchyme cells (Figure 2A’, upper arrow). In *Kif3a^−/−MM^* mice, KIF3A was lost in metanephric mesenchyme cells (box inset, Figure 2A”) but was expressed in ureteric cells (Figure 2A”, arrow in UB).

Next, we determined the effect of KIF3A deficiency on cilia. Cilia number was quantitated in sagittal tissue sections generated from the mid-point of the kidney by counting the number of cells, identified by DAPI, associated with a primary cilium, identified with anti-α-AcT. Deletion of *Kif3a* in ureteric or metanephric mesenchyme cells resulted in a gradual loss of primary cilia during embryogenesis. *Kif3a^−/−UB^* mice demonstrated 64% fewer ureteric-derived cells with primary cilia at E13.5 (Figure 2A’ versus 2A; % cells with primary cilia, *Kif3a^−/−UB^* versus WT: 33±3.46 versus 97±1.23, n = 5 mice/group) and 59% fewer cells with primary cilia at E15.5 (Figure 2B’ versus 2B; % cells with primary cilia, *Kif3a^−/−UB^* versus WT: 38±4.37 versus 97±1.07, n = 5 mice/group). By P0, primary cilia could be detected in collecting ducts only rarely (Figure 2D’ versus 2D). Furthermore, scanning electron microscopy (SEM) of collecting duct cells revealed decreased cilia number at E15.5 and complete absence of cilia at P0 (Figure 2F’ versus 2F, and 2G’ versus 2G, boxes). In *Kif3a^−/−MM^* kidneys, the number of metanephric mesenchyme cells with primary cilia was decreased by 70% at E13.5 (Figure 2A’’ versus 2A, asterisk; % cells with primary cilia, *Kif3a^−/−MM^* versus WT: 29±5.67 versus 99±1.14, n = 5 mice/group) and by 67% in proximal tubule cells at E15.5 (Figures 2C’’ versus 2C; % cells with primary cilia, *Kif3a^−/−MM^* versus WT: 32±3.09 versus 99±1.12, n = 5 mice/group). By P0, cilia were virtually absent from proximal tubule cells (Figure 2E” versus 2E). These results were confirmed by SEM analysis of proximal tubules, identified by apical brush border villae, at E15.5 and P0 (Figure 2H’’ versus 2H and 2I’’ versus 2I, boxes). Taken together, these results indicate that deletion of *Kif3a* results in a cell-specific gradual loss of primary ciliaduring embryonic kidney development with complete absence of cilia by P0.

**Figure 3:**
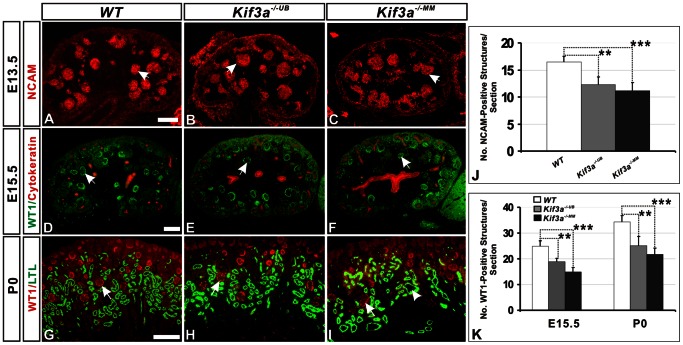
Nephron number is decreased in both *Kif3a^−/−UB^* and *Kif3a^−/−MM^* kidneys. (A–C) Nephrogenic precursor structures, marked by expression of NCAM (red color, marked by arrows), in *Kif3a^−/−UB^* mice (B) and *Kif3a^−/−MM^* mice (C) versus control mice (A). (D–F) Identification of glomeruli via expression of WT1 (green color, marked by arrow). Collecting ducts are identified by expression of cytokeratin (red color). (G–I) Identification of WTI-positive structures (red color, marked by arrow) at P0. Proximal tubules are identified by expression of LTL (green color, marked by arrowhead). (J, K) Quantification of the number of NCAM–positive and WT1-positive structures demonstrates a decreased number in *Kif3a^−/−UB^* and *Kif3a^−/−MM^* mice. (***, P<0.001; **, P<0.01; *, P<0.05). Scale bars: 25 micrometer.

*Kif3a^+/−UB^* and *Kif3a ^+/−MM^* heterozygote mice were viable and characterized by normal renal development (data not shown). In contrast, both *Kif3a^−/−UB^* and *Kif3a^−/−MM^* mice exhibited a remarkably similar histologic phenotype characterized by epithelial cysts (Figure S3H, I), a reduction in the number of NCAM-positive nephrogenic precursor structures (Figure 3A–3C), and glomeruli, which are characterized by expression of WT1 in podocytes (Figure 3D–I). As in previous analyses, quantitation of NCAM-positive and WT1-positive structures was performed in sagittal tissue sections generated starting at the mid-point of a kidney. The number of NCAM-positive structures in *Kif3a^−/−UB^* and *Kif3a^−/−MM^* mice was reduced by 25% and 32%, respectively, at E13.5 (Figure 3J, no. NCAM-positive structures/section, WT versus *Kif3a^−/−UB^*: 16.5±3.05 versus 12.33±2.36, p = 0.003; WT versus *Kif3a^−/−MM^* : 16.5±3.05 versus 11.16±2,47, p = 0.001, n = 4 mice/group). The number of WT1-positive structures in *Kif3a^−/−UB^* and *Kif3a^−/−MM^* mice was reduced by 24% and 34%, respectively, at E15.5 (Figures 3D–F and Figure 3K, no. WT1-positive structures/section: WT versus *Kif3a^−/−UB^*: 25±3.43 versus 18.8±2.71, p = 0.002; WT versus *Kif3a^−/−MM^*: 25±3.43 versus 15.71±1.25, p = 0.001, n = 4 mice/group), and by 25% and 35%, respectively, at P0 (Figure 3G–I and Figure 3K, no. WT1-positive structures/section: WT versus *Kif3a^−/−UB^*: 34.25±5.32 versus 25.12±3.68, p = 0.002; WT versus *Kif3a^−/−MM^*: 34.25±5.32 versus 21.6±5.69, p = 0.0001, n = 4 mice/group). Nephron number was also quantitated by counting the number of glomeruli, identified by their characteristic morphology in sagittal tissue sections generated in both directions from the mid-point of a kidney at 36 micrometer intervals to the outer limit of the organ [17]. At E15.5, this analysis demonstrated a reduction in glomerular number of 24% in *Kif3a^−/−UB^* kidneys and 33% in *Kif3a^−/−MM^* kidneys (Figure S3B and S3C versus S3A and Figure S3J; no. glomeruli/kidney, WT versus *Kif3a^−/−UB^*: 78.14±7.81 versus 62.85±4.29, p = 0.002; WT versus *Kif3a^−/−MM^* : 78.14±7.81 versus 54.85±7.20, p = 8.75E–05, n = 7 mice/group). Glomerular number was similarly reduced at E18.5 (Figure S3D–F, Figure S3J; no. glomeruli/kidney, WT versus *Kif3a^−/−UB^*: 1153±151 versus 887±74, p = 0.01; WT versus *Kif3a^−/−MM^*: 1153±151 versus 827±169, p = 0.003, n = 6 mice/group) and at P0 (Figure S3G–I, Figure S3J; no. glomeruli/kidney, WT versus *Kif3a^−/−UB^*: 2025±211 versus 1513±290, p = 0.01; WT versus *Kif3a^−/−MM^*: 2025±211 versus 1285±255, p = 0.001, n =  mice/group). Cyst formation in glomeruli and tubules (Figure 3I and Figure S3H and S3I, arrowheads) was consistent with published analysis of KIF3A function in the kidney [26]. Together, these results demonstrate that *Kif3a* deficiency in either the ureteric or metanephric mesenchyme cell lineage causes nephron deficiency.

### *Kif3a* Controls Branching Morphogenesis in a GLI3R-dependent Manner

**Figure 4:**
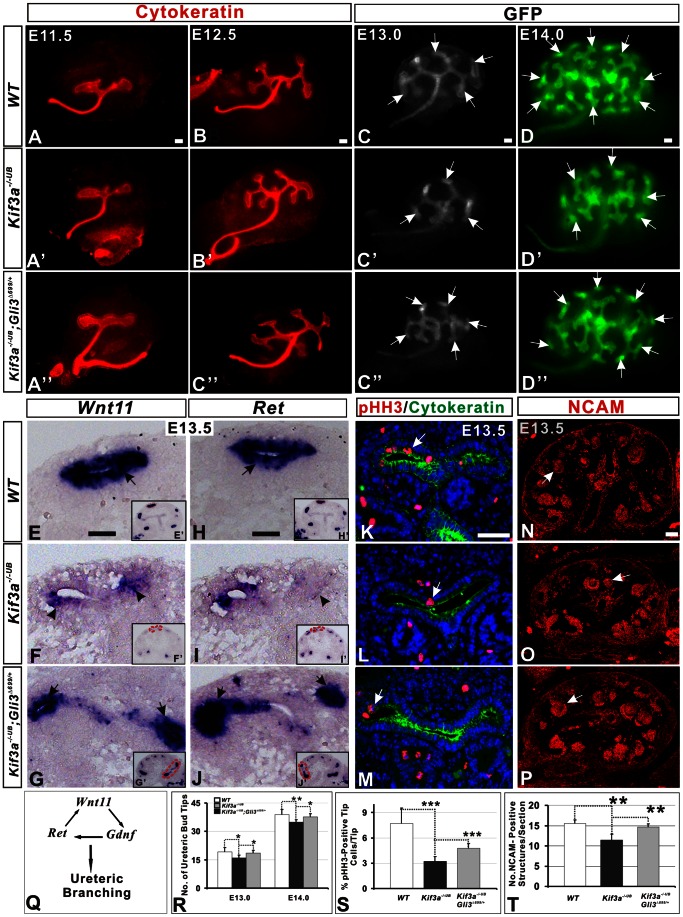
*Kif3a* controls renal branching morphogenesis in a GLI3R-dependent manner. (A–C’’) Cytokeratin immunofluorescence demonstrating ureteric branching at E11.5 and E12.5. The branch pattern is similar among *WT* (A,B), *Kif3a^−/−UB^* (A’,B’) and *Kif3a^−/−UB^*;*Kif3a^Δ699/+^*(constitutive expression of *Gli3^Δ699/+^* in *Kif3a^−/−UB^* background, A’’, B’’) kidneys. (C–D’’) Ureteric branching in *WT*, *Kif3a^−/−UB^*, and *Kif3a^−/−UB^*;*Gli3^Δ699/+^*mice at E13.0 and E14.0. In *Hoxb7-GFP-Cre* mice, GFP expression in the kidney is restricted to the ureteric cell lineage. Ureteric branches expressing GFP are visualized in whole mount preparations of kidney explants generated from E13.0 (C, C’ and C’’) and E14.0 (D, D’ and D’’). Imaging suggests that branch number in *Kif3a^−/−UB^* mice (C’, D’) is less than that in *WT* (C, D) and *Kif3a^−/−UB^*;*Gli3^Δ699/+^*mice (C’’, D’’). (E–J) *Wnt11* and *Ret* expression. In WT kidney, *Wnt11* (E) and *Ret* (H) are strongly expressed in ureteric tip cells (arrows). Expression of *Wnt11* (F) and *Ret* (I) is markedly decreased in *Kif3a^−/−UB^* mice. In *Kif3a^−/−UB^*;*Gli3^Δ699/+^* mice, *Wnt11* (G) and *Ret* (J) expression are rescued to levels similar to that observed in *WT* mice. (K–M) Phosho-Histone H3 (pHH3) is stained in mitotic cell of ureteric tip (red color) that is marked by cytokeratin (green color) at E13.5. (N–P) NCAM staining showing nephrogenic structures at E13.5. Decreased formation of NCAM-positive structures in *Kif3a^−/−UB^* mice (O versus N) is rescued in *Kif3a^−/−UB^*;*Gli3^Δ699/+^* (P versus O) mice (Q) Schematic showing the *Wnt11/Gdnf/Ret* gene network that promotes ureteric branching morphogenesis. (R) Quantitation of ureteric branch tip number reveals a significant decrease in *Kif3a^−/−UB^* mice compared to WT at E13.0 and E14.0 but increased branching in *Kif3a^−/−UB^*;*Gli3^Δ699/+^* mice compared to *Kif3a^−/−UB^* mice. (S) Quantitation of mitotic tip cells in K, L and M. pHH3 marked cells are decreased in *Kif3a^−/−UB^* mice (L) compared to WT (K). pHH3-positive cells is remarkably increased in in *Kif3a^−/−UB^*;*Gli3^Δ699/+^* mice compared to *Kif3a^−/−UB^* mice (M versus L). (T) Quantitation of NCAM-positive structures in N, O and P reveals a significant decrease in *Kif3a^−/−UB^* mice compared to WT and *Kif3a^−/−UB^*;*Gli3^Δ699/+^* mice. (*, P<0.05, **, P<0.01, *** P<0.001). Scale bars: 50 micrometer.

Formation of nephrons is initiated by signals released from ureteric bud-derived cells adjacent to mesenchymal nephrogenic progenitor cells [15]. Since nephron number is directly related to the number of ureteric branches elaborated during branching morphogenesis, we analyzed the effect of *Kif3a* deficiency on formation of ureteric branches by quantitating the number of ureteric bud tips, marked by expression of either GFP or Cytokeratin. In *Kif3a^−/−UB^* mice, formation of the initial ‘T’ shape ureteric branch at E11.5 and the first two branch generations at E12.5 was normal compared with WT (Figure 4A, 4A’, 4B and 4B’). However, at E13.0 and E14.0, the number of ureteric bud tips was significantly reduced in *Kif3a^−/−UB^* mice (Figures 4C’ versus 4C, 4D’ versus 4D and Figure 4R; no. UB tips/kidney, WT versus *Kif3a^−/−UB^* at E13.0∶19.2±2.28 versus 16±1.41, p = 0.03; WT versus *Kif3a^−/−UB^* at E14.0∶39±2.59 versus 34±1.15, p = 0.03,n = 6 mice/group). Since ureteric branching is dependent, in part, on ureteric tip cell proliferation, we next identified ureteric tip cells undergoing mitosis using antibody specific for phospho-histone H3. Consistent with the decrease in ureteric branching, mitotic ureteric tip cell was decreased in *Kif3a^−/−UB^* mice (Figure 4K, 4L, and 4S; no. mitotic tip cells/tip, WT versus *Kif3a^−/−UB^*: 7.72±1.81,versus 3.23±0.54, p = 0.001, n = 5 mice/group). These results are consistent with decreased nephron number observed in *Kif3a^−/−UB^* mice (Figure 3B versus 3A, and Figure 3J).

Ureteric branching is controlled by a *Wnt11/Ret/Gdnf* signaling axis [27], [28]. GDNF, an extracellular ligand expressed by metanephric mesenchyme cells, binds to RET on the surface of ureteric tip cells. In turn, GDNF/RET signaling controls ureteric tip cell expression of *Wnt11*, a positive regulator of ureteric branching (Figure 4Q). In *Kif3a^−/−UB^* mice, overall expression of *Wnt11* and *Ret* was markedly reduced (Figures 4F’ and 4I’, inserts). Higher resolution imaging of ureteric tips revealed weak expression of *Wnt11* and *Ret* in ureteric cells (Figure 4F and 4I, arrowheads).

Previously, we demonstrated that expression of *Wnt11* and *Ret* in ureteric tip cells is controlled by GLI3R [17]. Suppression of GLI3R formation in *Patched1* deficient mice decreases *Wnt11* and *Ret* expression and lowers ureteric branch and nephron number. Obligate expression of GLI3R via the *Gli3^Δ699^* allele, which expresses GLI3R in a constitutive manner [29], rescues these abnormalities [17]. Since defects in the primary cilium alter the ratio of GLI3 activator to GLI3R in favor of GLI3 activator [1], we hypothesized that GLI3R deficiency in *Kif3a^−/−UB^* mice could cause reduced branching morphogenesis. Thus, we determined whether constitutive expression of GLI3R in *Kif3a^−/−UB^* mice rescues ureteric branching. Analysis of *Kif3a^−/−UB^*;*Gli3^Δ699/+^* mice at E13.0 and E14.0 revealed that the number of ureteric tips was significantly increased compared to *Kif3a^−/−UB^* mice (Figure 4C’’, 4D’’ and 4R; no. UB tips/kidney, *Kif3a^−/−UB^*;*Gli3^Δ699/+^* versus *Kif3a^−/−UB^*, at E13.0∶19.2±2.28 versus 15.33±0.82, p = 0.01; at E14.0∶40.0±2.33 versus 33.33±2.5, p = 0.001, n = 6 mice/group). Further, expression of *Wnt11* and *Ret* was markedly increased compared to that observed in *Kif3a^−/−UB^* mice (Figure 4G and 4J versus Figure 4F and 4I). Concomitant with a rescue of ureteric branching, the number of NCAM-positive nephrogenic precursors in *Kif3a^−/−UB^*;*Gli3^Δ699/+^* mice was comparable to that observed in *WT* mice (Figure 4P versus 4O, and 4T; NCAM-positive structures/section - *Kif3a^−/−UB^*;*Gli3^Δ699/+^* versus WT: 15.5±3.40 versus 14.7±4.24, P  = 0.27, n = 4 mice/group). Taken together, these results demonstrate that *Kif3a* regulates branching morphogenesis in a GLI3R-dependent manner.

### *Kif3a* Controls Metanephric Mesenchyme Cell Survival

**Figure 5:**
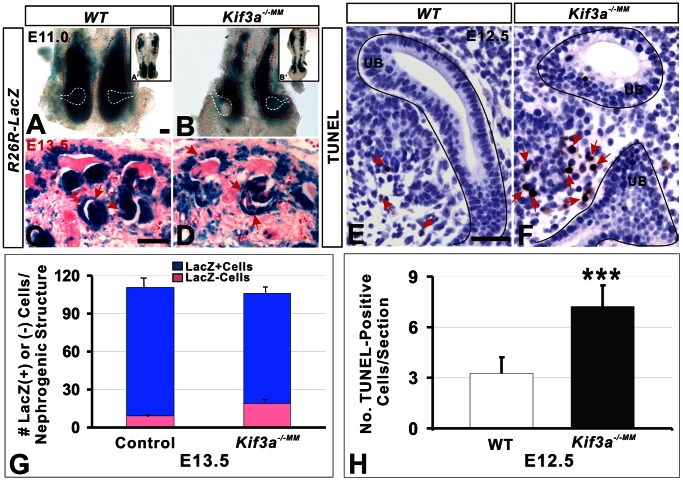
*Kif3a* controls survival of nephrogenic precursor cells. (A–D) LacZ expression in kidney tissue. (A, B) LacZ-marked metanephric blastema at E11.0 is demarcated by red dotted lines. The position of the ureteric bud is marked by white dotted lines. The metanephric blastema is smaller in *Kif3a^−/−MM^* mice (B) compared to control (A). (C, D) Incorporation of pink (lacZ-negative; *Kif3a^+/+^* cells) into nephrogenic structures (arrows) is greater in *Kif3a^−/−MM^* mice (D) compared to controls (C). (E, F) TUNEL assay identify the apoptotic cells (arrows) in the metanephric mesenchymal cells at E12.5. The number of TUNEL-positive cells is greater in *Kif3a^−/−MM^* mice (F, arrows) compared to control (E, arrows). (G, H) Quantitation of lacZ-positive and –negative cells (G) and TUNEL-positive cells (H). *Kif3a^−/−MM^* mice exhibit a significantly larger contribution of lacZ-negative cells to nephrogenic structures (D), and increased apoptosis in metanephric mesenchymal cells (F). (***, P<0.001; **, P<0.01; *, P<0.05). Scale bars: 50 micrometer.

Decreased nephron number in *Kif3a^−/−MM^* mice (Figure 3J, 3K and Figure S3J) demonstrated that *Kif3a* functions in a cell autonomous manner within metanephric mesenchyme. We investigated mechanisms underlying *Kif3a-*dependent functions by first analyzing the effect of *Kif3a* deficiency on metanephric mesenchyme cells that are progressively committed to a nephrogenic fate. *Kif3a^loxP/loxP^* [3] mice were intercrossed with *R26R^LacZ/LacZ^* [30] to generate *Kif3a^loxP/loxP^;R26R^Lacz/+^* mice, which were used as a reporter for *Rarb2-Cre* activity and to label *Kif3a*-deficient cells [31]. Analysis of *lacZ* expression at E11.0 demonstrated that the metanephric blastema was smaller in *Kif3a^−/−MM^* mice compared to controls (Figure 5A and 5B, area of *lacZ*-positive tissue (µm^2^): control –53806.86±662; *Rarb2-Cre;R26R;Kif3a^loxP/−^* –33486±2563; p = 3.27×10^−5^, n = 6 mice/group). Consistent with this finding, the number of SIX2-positive nephrogenic progenitor cells was decreased in *Kif3a^−/−MM^* mice (Figure S4D versus S4E, S3F; no. SIX2-positive cells/tissue section, WT versus *Kif3a^−/−MM^* : 48.5±2.65 versus 43.25±3.5, p = 0.008, n = 6 mice/group). This finding is consistent with the decreased number of NCAM-positive structures in *Kif3a^−/−MM^* mice (Figure 3A, 3C and 3J). Next, we determined whether these *Kif3a*-dependent effects on nephron number were associated with altered cell proliferation and/or apoptosis. We analyzed apoptosis at the stages when the process of nephron formation is well established (E12.5). The number of apoptotic nuclei, identified by the TUNEL assay, was significantly increased in metanephric mesenchyme cells in *Kif3a^−/−MM^* kidney tissue (Figure 5F versus 5E and 5H; no. TUNEL-positive cells/tissue section, WT versus *Kif3a^−/−MM^*: 3.25±0.96 versus 7.2±1.29, p = 0.0006, n = 4 mice/group). In contrast, the proportion of proliferating (BrdU-positive) cap mesenchyme cells did not differ in mutant mice (Figure S4A, S4B and S4C) (% BrdU-positive cap mesenchyme cells, WT versus *Kif3a^−/−MM^*: 89.6±2.88 versus 88.2±3.56, p = 0.51, n = 5 mice/group ). Previously, we demonstrated that deficiency of nephrogenic progenitors during early stages of murine kidney development can limit the number of nephrogenic cells available to participate in more advanced stages of nephron formation [31]. We investigated this possibility in *Kif3a^−/−MM^* mice using the R26R allele and examining cortical sections for *lacZ* expression in nephrogenic structures. In control mice, *lacZ*-marked cells comprised 95% of the total number of cells (4547 of 4613 cells) resident within nephrogenic structures. In contrast, in mutant mice, *lacZ*-marked cells constituted only 82% of the total number of cells in nephrogenic structures (2413 of 3251 cells) (p = 0.02; n = 6 cortical section counted in each of 6 mice/group, Figure 5C, 5D and 5G). These data suggest that depletion of *Kif3a* deficient nephrogenic progenitors may provide a selective advantage for *Rarb2-Cre-*negative cells with WT levels of KIF3A to participate in nephron formation. Together, these analyses of cell fate and cell survival indicate that *Kif3a* deficiency interferes with survival of nephrogenic progenitor cells and decreases the size of the cellular pool available to participate in nephron formation.

### *Kif3a* Deficiency Leads to *Fgf8* Deficiency in Nephrogenic Mesenchyme

**Figure 6:**
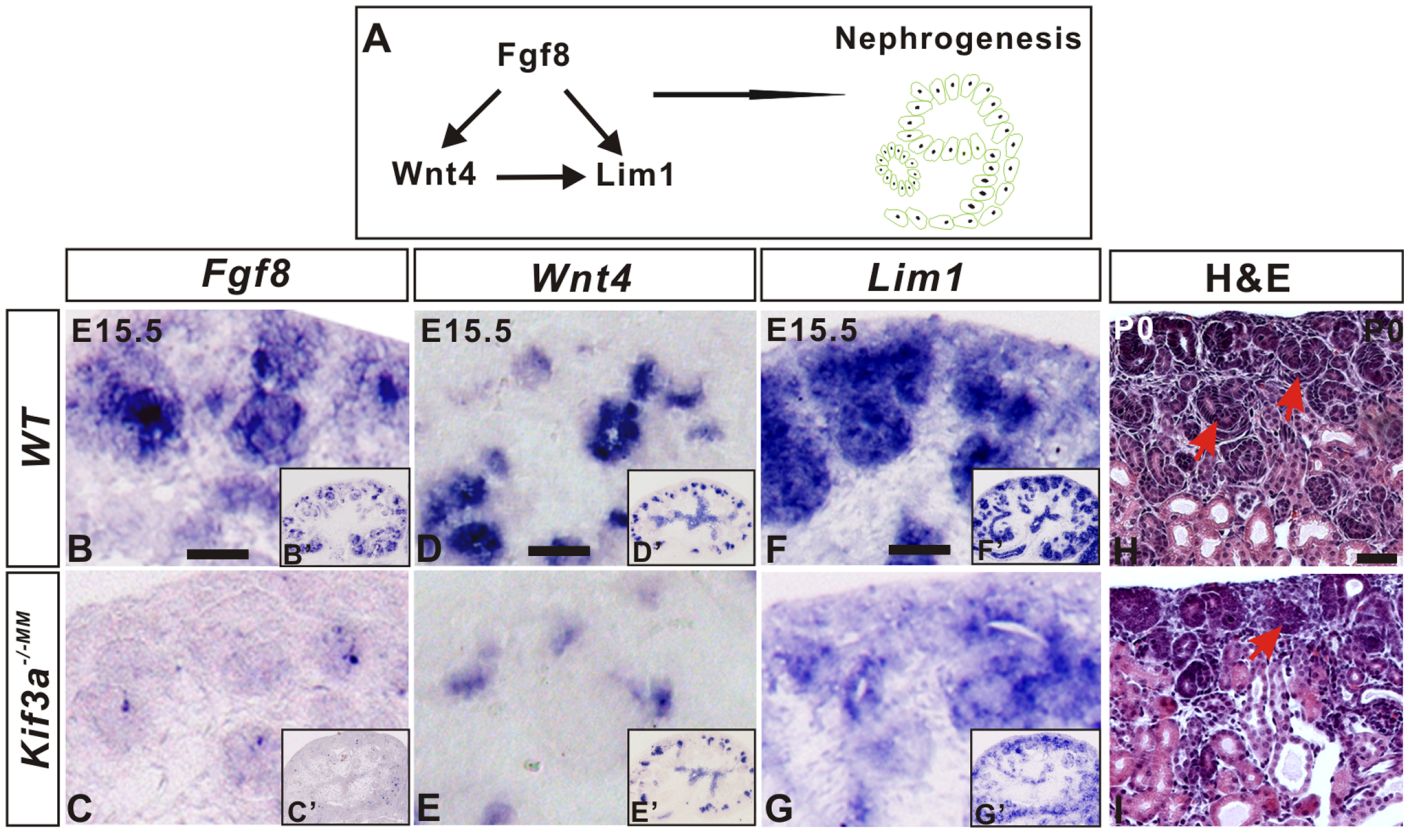
*Kif3a* controls expression of a *Fgf8*-mediated signaling pathway during nephron formation. A. Schematic of *Fgf8/Wnt4/Lim1* signaling pathway required during nephron formation. (B–G) *In situ* RNA hybridization in kidney tissue sections generated from E15.5 embryos. *Fgf8* (B, C), *Wnt4* (D, E) and *Lim1* (F, G) are expressed in nephrogenic progenitors in WT mice. *Fgf8* expression is barely detectable in *Kif3a^−/−MM^* mice (C, C’). Expression of *Wnt4* (E) is markedly decreased as is expression of *Lim1* (G). (H, I) Histological sections from P0 kidney tissue. There are fewer S-shaped bodies (arrows) in *Kif3a^−/−MM^* mice (I) than in control mice (H). Scale bars: 25 micrometer.

**Figure 7:**
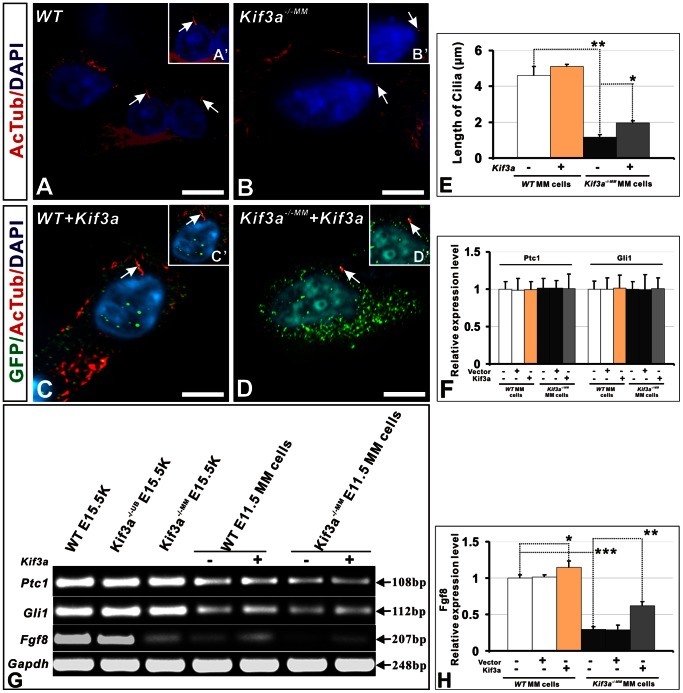
*Kif3a* acts upstream of *Fgf8*. (A–D) Analysis of primary cilia (arrows) in metanephric mesenchyme cells derived from *WT* and *Kif3a^−/−MM^* metanephroi dissected free of ureteric bud. Cilia are identified by expression of α-AcT. (A’–D’) Higher magnification of images in A–D, respectively. WT and *Kif3a*-deficient cells were transfected with a plasmid encoding *Kif3a* fused to GFP. Cilia in *Kif3a*-deficient mesenchyme cells (B, B’) are vestigial in comparison to cilia on WT cells (A, A’). Transfection with *Kif3a* results in localization of GFP to the cilium in each treatment group (C, D) and lengthening of the cilium in *Kif3a^−/−MM^* cells (D versus B). (E) Quantitation of cilia length in untransfected and transfected WT and *Kif3a^−/−MM^* cells. Expression of *Kif3a* partially rescues cilia length in *Kif3a-*deficient cells. (F) Quantitation of *Ptc1* and *Gli1* mRNA expression, measured by quantitative RT-PCR in untransfected and transfected WT and *Kif3a^−/−MM^* cells. *Ptc1* and *Gli1* mRNA expression is not affected by *Kif3a* deficiency or transfection with *Kif3a.* (G) Expression of *Fgf8*, *Ptc1*, and *Gli1* mRNA, measured by real time RT-PCR using RNA isolated from kidney explants and from untransfected and transfected cultured metanephric mesenchyme cells. (H) Quantitation of *Fgf8* mRNA levels measured by quantitiative RT-PCR as in panel G. MM, metanephric mesenchyme; UB, ureteric bud; WT, wild-type. (***, P<0.001; **, P<0.01; *, P<0.05), Scale bars: (A–D) 25 micrometer, (I–L) 50 micrometer.

Nephron formation is dependent on a signaling axis in which *Fgf8* functions upstream of *Wnt4* and *Lim1* (Figure 6A). In the absence of *Fgf8,* neither *Wnt4* nor *Lim1* is expressed and nephron formation largely fails to progress to the stage of the S-shaped body [22]. Since FGF receptors have been localized to the cilium in nonrenal tissues [12], we investigated the possibility that *Kif3a* deficiency and loss of primary cilia interfere with FGF8-mediated signaling during nephron formation. In support of this possibility, analysis of kidney tissue derived from *Kif3a^−/−MM^* mice at P0 revealed a 28% reduction in the number of S-shaped bodies (Figure 6H and 6I, *Kif3a^−/−MM^* versus control – no. S-shape bodies/kidney: 6.16±0.5 versus 3.83±1.73; p = 0.0003, n = 5 mice/group). While expression of *Fgf8* mRNA was only mildly decreased at E13.5 (Figure S5), by E15.5, *Fgf8* expression was almost undetectable (Figure 6C versus 6B, insert and Figure 7G, lane 3). Further, expression of *Wnt4* and *Lim1* was markedly reduced with only a focal pattern of expression within some nephrogenic structures (Figure 6E versus 6D and 6G versus 6F, insert). Together, these results indicate that *Kif3a* deficiency decreases *Fgf8-*dependent signaling during renal development.

### *Kif3a* Controls Expression of *Fgf8* by Metanephric Mesenchyme Cells

Previous studies have demonstrated that *Fgf8* signalling regulates cilia length via the IFT pathway in diverse epithelia [11]. Yet, a role for *Kif3a* in controlling *Fgf8* expression has not been previously elucidated. Our results demonstrating that *Kif3a* deficiency precedes *Fgf8* deficiency in metanephric mesenchyme suggested the possibility that *Kif3a* controls *Fgf8* expression in metanephric mesenchyme cells. We tested our hypothesis in cultured metanephric mesenchyme cells isolated from WT and *Kif3a^−/−MM^* mice and transfected with a DNA construct encoding a *Kif3a-*GFP fusion protein (Figure 7). Analysis of primary cilia length, identified by α-AcT expression and imaged by confocal microscopy in cultured primary metanephric mesenchyme cells, revealed that cilia were markedly shorter in cells isolated from *Kif3a^−/−MM^* mice (Figure 7B versus 7A and Figure 7E, cilium length, WT versus *Kif3a^−/−MM^* MM cells: 4.585±1.523 micrometer versus 1.576±0.449 micrometer; p = 3.5E–10, n  = 10 culture wells/group). Transfection of *Kif3a* fused with GFP in metanephric mesenchyme cell cultures caused localization of KIF3A and GFP to primary cilia and a significant increase in cilia length in metanephric mesenchyme cells isolated from *Kif3a^−/−MM^* mice (Figure 7C and 7D and Figure 7E, cilium length, untransfected versus transfected *Kif3a^−/−MM^* metanephric mesenchyme cells: 1.576±0.449 micrometer versus 1.962±0.360 micrometer; p = 0.03, n = 10 culture wells/group). Next, we analyzed the effect of *Kif3a* on expression of Hh signaling effectors, the expression of which has been shown to be dependent on *Kif3a* in nonrenal tissues. Surprisingly, neither loss of *Kif3a* expression in *Kif3a^−/−MM^* metanephric mesenchyme tissue, cultured metanephric mesenchyme cells nor transfection-mediated *Kif3a* expression in these cells was associated with a detectable change in *Ptc1* or *Gli1* mRNA levels assayed by quantitative PCR (Figure 7G and 7F). In contrast, *Fgf8* mRNA expression was significantly lower in cultured *Kif3a^−/−MM^* metanephric mesenchyme cells compared to controls (Figure 7G and 7H). Moreover, *Kif3a* transfection significantly increased expression of *Fgf8* mRNA in both WT and *Kif3a-*deficient metanephric mesenchyme cells (Figure 7G and 7H). Together, these data indicate that *Kif3a* controls *Fgf8* expression in metanephric mesenchyme cells.

## Discussion

**Figure 8:**
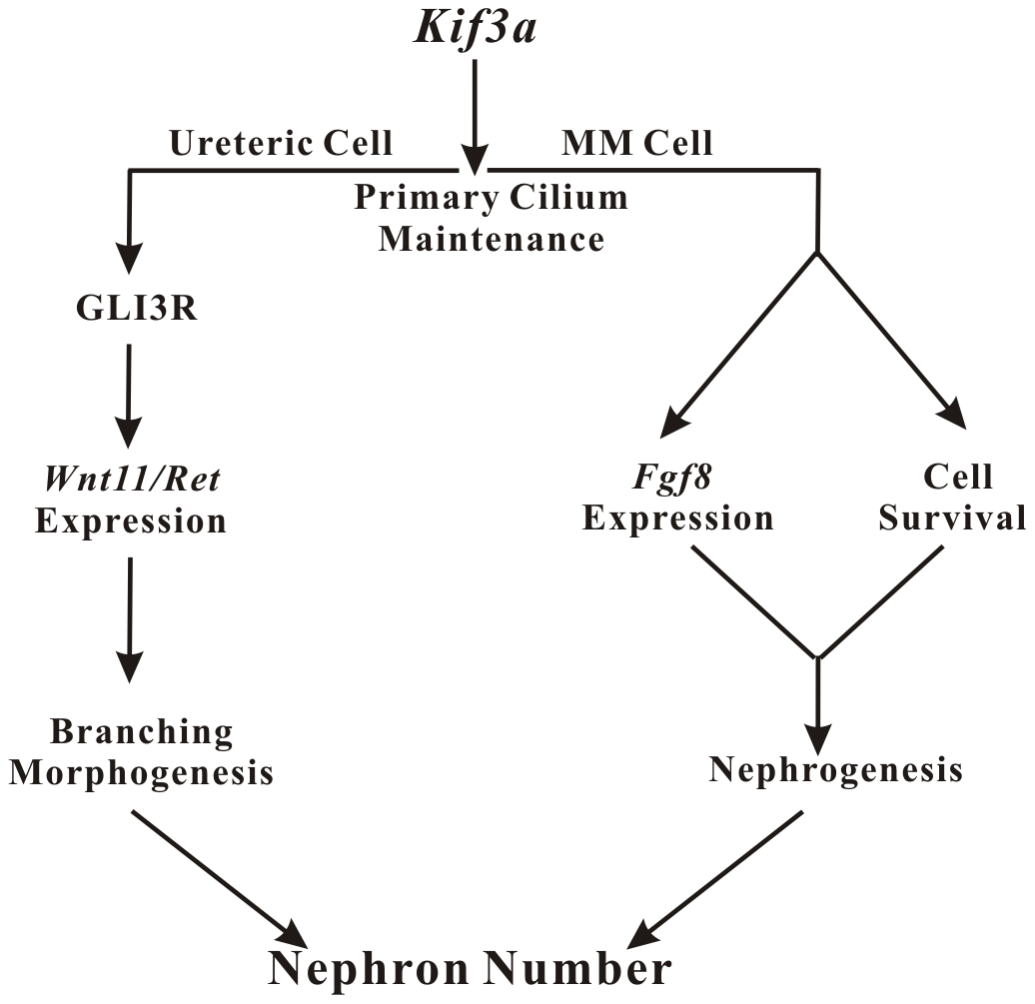
Model of *Kif3a-*mediated regulation of nephron number. A *Kif3a* control is required in both ureteric and metanephric mesenchyme cells for processes that control nephron number. In ureteric cells *Kif3a* acts in a GLI3R-dependent manner to control expression of *Wnt11/Ret* and ureteric branching. In metanephric mesenchyme cells, *Kif3a* functions to control cell survival and expression of *Fgf8*, which is required for maturation of nephrogenic progenitors. The lineage-specific functions of *Kif3a* in ureteric and metanephric mesenchyme cells converge to control nephron number.

Cilia proteins KIF3A, IFT88 and IFT20, which are involved in IFT [2], [32], [33], are required for renal ciliogenesis; inactivation of each is known to cause cystic kidney disease [3], [14], [34], [35]. To our knowledge this is the first study demonstrating a role for the primary cilium in the regulation of nephron number. Our data show that *Kif3a* expression and primary cilia are found in both ureteric cells and metanephric mesenchyme cells from the onset of murine kidney development. CRE-mediated recombination using a *Kif3a^loxP^* allele results in near total loss of *Kif3a* in CRE-expressing cells. Loss of cilia occurs with slower kinetics. Yet, *Kif3a* deficiency and a decrease in the number of primary cilia reduce the number of nephron precursor structures formed and the final number of mature nephrons. Experiments that investigated the mechanisms underlying this phenotype support a model of *Kif3a* function during murine renal development (Figure 8). Our model suggests that the functions of *Kif3a* in controlling nephron formation are specific to the ureteric and metanephric mesenchyme cell lineages. In the ureteric lineage, *Kif3a* controls the number of ureteric branches formed in a GLI3R-dependent manner. Control of branch number is a critical determinant of nephron number. In metanephric mesenchyme cells, *Kif3a* exerts two major distinct effects. First, *Kif3a* controls cell survival such that the metanephric blastema that gives rise to nephrons is smaller in *Kif3a* deficient mice and that surviving *Kif3a* deficient metanephric mesenchyme cells are less able to take part in forming nephrogenic structures compared to their wild type counterparts. Second, *Kif3a* controls expression of genes required during nephron formation in metanephric mesenchyme cells.

Our results demonstrate a central role for KIF3A in controlling ureteric and mesenchyme cell function. Lineage-specific deletion of *Kif3a* is efficient with little residual *Kif3a* mRNA expression in either the ureteric or metanephric mesenchyme cell populations by E11.5, the stage at which these cell populations were separated and analyzed in *Kif3a^−/−UB^* and *Kif3a^−/MM^* mice (Figure S2). Our results also strongly suggest that the functions of KIF3A protein are related to its specific expression in the primary cilium. Analysis of KIF3A protein expression in *Kif3a-*deficient mouse strains demonstrated a specific KIF3A signal in the primary cilium (Figure 2A, 2A’, 2A’’). Interestingly, primary cilia exhibit a comparatively slower turnover rate, compared to KIF3A protein, in ureteric and mesenchyme cells. While we could detect very little KIF3A protein by E13.5, in the ureteric or metanephric mesenchyme cells of *Kif3a^−/−UB^* and *Kif3a^−/MM^* mice, respectively, primary cilia could be detected at E15.5, albeit in reduced numbers. These observations suggest that primary cilium structure can be maintained in non-dividing cells in the face of KIF3A depletion. However, our studies do not provide information as to whether the function of KIF3A-deficient cilia is normal. Our data are also consistent with the rather modest effect of KIF3A deficiency on nephron number. Given the slow kinetics of KIF3A depletion and loss of cilia, it is likely that remaining number of cilia during early critical stages of nephron formation are sufficient to support this process.

Our results demonstrate a critical role for GLI3R in primary cilium function in ureteric cells and are consistent with our previous work related to GLI3R and ureteric branching [17]. The initial stage of ureteric bud invasion into the mesenchyme appears normal in *Kif3a^−/−UB^* kidneys. However, the expression of *Wnt11* and *Ret,* both of which are required for ureteric branching [28], [36], is decreased in mutant kidneys at E13.5 (Figure 4F,G and 4H, I). Primary cilia are present on only a subset of UB cells at this time point as primary cilia are gradually lost from E13.5 to P0. Concomitantly, the expression of the ureteric tip markers, and the number of ureteric tips is significantly reduced at E14.0.

In our previous published work, analysis of Hh signaling activity, using a *Ptc1-lacZ* reporter, demonstrated that ureteric tips are characterized by low Hh activity [17]. Activation of Hh signaling activity in ureteric cells in mice with *Ptc1* deficiency causes decreased *Ret* and *Wnt11* expression, decreased ureteric branching and low nephron number. But, constitutive expression of GLI3R (via the *Gli3* ^Δ*699*^ allele) rescues these abnormalities [17] and suggests that GLI3R, rather than GLI activators, is the regulatory target of SHH signaling during formation of nephrons. Our prior analyses in *Shh* deficient mice also support the concept that regulation of GLI3R is the critical event during kidney development. Mice with homozygous deficiency of *Shh* are characterized by disruption of initial ureteric-metanephric mesenchyme tissue interactions and an elevated ratio of GLI3R to GLI activator proteins in *Shh* deficient renal tissue. Remarkably, these abnormalities are rescued by homozygous deficiency of *Gli3* in *Shh* deficient mice, thus implicating regulation of GLI3R formation as a critical event during renal development [16]. Results here suggest that the primary cilium plays a critical role in GLI3R expression in ureteric cells.

In contrast to ureteric cells, our results do not invoke Hh signaling and GLI3R in regulating metanephric mesenchyme cell survival and nephron formation. Our data, are consistent with our published analysis of kidney development in mice with conditional inactivation of *Smo* in metanephric mesenchyme cells [37]. In these mice (*Rarb2-Cre;Smo^loxP/−^*), genetic inactivation of *Smo* was mediated by CRE recombinase, the expression of which was driven by a *Rarb2* promoter element which directs expression in the intermediate and metanephric mesenchyme [31]. In *Rarb2-Cre;Smo^loxP/−^* mice, renal development is normal until E15.5 when pelvic dilatation arises due to ureteric dyskinesia and abnormal pacemaker cell function, demonstrating that loss of Hh signaling in intermediate and metanephric does not disrupt the mass of cells available to take part in nephron formation. Our results in cultured metanephric mesenchyme cells (Figure 7) are consistent with these findings in *Rarb2-Cre;Smo^loxP/−^* mice since *Ptc1* and *Gli1* are expressed in *Kif3a* deficient metanephric mesenchyme cells isolated at E11.5. Moreover, transfection of *Kif3a* in *Kif3a-*deficient cells has no significant effect on *Ptc1* and *Gli1* expression.

Our analyses in *Kif3a^−/−MM^* mice and in cultured metanephric mesenchyme cells suggest a role for KIF3A upstream of FGF8. *Kif3a^−/−MM^* mice are characterized by cilia in developing nephron structures and intact nephron formation before E13.5. However, the total pool of *Kif3a*-negative mesenchymal precursor cells is decreased in the kidney blastema at E11.0. Surviving *Kif3a*-negative mesenchyme cells exhibit the ability to undergo a mesenchymal to epithelial transition probably because a sufficient level of *Fgf8, Wnt4* and *Lim1* mRNA is present to support this process (Figure S5). However by E15.5, *Fgf8* expression is lost – the same stage at which the number of cilia is significantly reduced in mesenchyme- derived cells. By E15.5, *Wnt4* and *Lim1* expression is markedly reduced consistent with loss of *Fgf8*. Yet, our results related to FGF8 are distinct from previous published analyses of *Fgf8* activity during renal development. Mice with total loss of *Fgf8* are able to initiate formation of nephrons but development of nephron precursors does not progress to the S-shape stage. While *Kif3a^−/−MM^* mice similarly exhibit a lower number of S-shape bodies compared to controls, S-shaped bodies are formed [22]. In FGF8-deficient mice, cells within nascent nephrons undergo high rates of apoptosis [22], a finding that is similar to that we observed in *Kif3a^−/−MM^* mice. Thus, decreased expression of *Fgf8* may be the cause of increased cell death in *Kif3a^−/−MM^* null kidneys. Our studies in cultured WT and *Kif3a-*deficient metanephric mesenchyme cells further link *Kif3a* to *Fgf8.* Previous studies demonstrated that *Fgf* signaling regulates cilia length through *Fgf8-Fgfr1* and the IFT pathway [11]. Our data show that *Fgf8* expression is decreased in *Kif3a-*deficient cells and that *Fgf8* expression is partially restored to WT levels by transfection of these cells with a plasmid encoding *Kif3a.*

## Materials and Methods

### Mouse Strains and Genotyping

The following mouse strains were used in these studies: Kif3a^loxP/loxP^, Kif3a^+/−^, Cre-ER™, Cre-ER™;Kif3a^loxP/−^ (termed Kif3a™), Hoxb.7-CreEGFP;Kif3a^+/−^, Rarb2-Cre;Kif3a^+/−^, Hoxb.7-CreEGFP;Kif3a^loxP/−^ (termed Kif3a^−/− UB^), Rarb2-Cre;Kif3a^loxP/−^ (termed Kif3a^−/− MM^), Hoxb.7-CreEGFP;Kif3a^loxP/+^ (termed Kif3a^+/− UB^); Rarb2-Cre;Kif3a^loxP/+^ (termed Kif3a^+/− MM^), Hoxb.7-CreEGFP;Kif3a^loxP/−^;Gli3^Δ699/+^(termed Kif3a^−/− UB^;Gli3^Δ699/+^) and R26R^LacZ/LacZ.^;Kif3a^flox/flox^ [3] mice were maintained on the C57BL/6 (B6) genetic background. Cre-ER™ [23], Hoxb7-CreEGFP [24] and Rarb2-Cre [25] mice were maintained on the CD1 inbred genetic background. Cre-ER™;Kif3a^+/−^, Cre-ER™;Kif3a^flox/−^(Kif3a™); Hoxb7-CreEGFP;Kif3a^+/−^; Hoxb7-CreEGFP;Kif3a^flox/−^ (Kif3a^−/−UB^) and Rarb2-Cre;Kif3a^+/−^, Rarb2-Cre;Kif3a^flox/−^ (Kif3a^−/−MM^) mice were maintained on a mixed background. R26R reporter mice [30] were maintained on a B6 x129/SV mixed genetic background. Genotyping was performed by PCR using genomic DNA isolated from ear clips or tails. PCR primers are listed in Table S1 in File S1.

### Tamoxifen-induced CRE Recombinase Expression

Tamoxifen (TM, T5648, Sigma) was dissolved in sesame oil at a concentration of 20 mg/ml. TM was injected at a dose of 3mg/40g body weight intraperitoneally into pregnant mice at E10.5.

### Tissue-based Assays

Non-radioactive section *in situ* hybridization was performed as per published methods using 5µm paraffin-embedded tissue sections [38]. Digoxigenin-labeled antisense probes were generated from linearized plasmids containing mouse *Wnt4* [39], *Wnt11* [40], *Wnt9b* [20], *Ret* [41], *Foxd1* [42], *Lim1* [25], *Fgf8* [43], [44]. To detect LacZ activity, tissues were fixed in 2% paraformaldehyde (PFA), embedded in OCT (Tissue-TeK 4583), and cryosectioned at 10 micrometer. Sections were dried at RT for 30 minutes, rinsed in PBS, and stained with standard X-gal staining solution overnight at 37°C before counterstaining with Eosin. The number of NCAM-positive structures, WT1-positive structures and S-shaped bodies was estimated by counting the number of structures in five tissue sections - a mid-sagittal section and two sections generated 40 micrometer and 80 micrometer in both directions from the mid-sagittal section - in each kidney analyzed. Quantitation was expressed the number of structures per section [43]. The average number of mitotic cell of ureteric tip (mitotic cell number/tip) was quantified by counting in five randomly chosen ureteric tips per section; five sections in each kidney were analyzed. The terminal deoxynucleotidyl transferase**-**mediated dUTP nick-end labelling (TUNEL) method was used to detect apoptosis. Paraffin wax was removed from 5 µm tissue sections with xylene and tissues were rehydrated and incubated with 20 mg/ml of Proteinase K for 15 minutes at 37°C. Non-specific binding was reduced with a hydrogen peroxide/methanol solution. Fragmented DNA was labelled using a reaction mixture, according to the manufacturer’s instructions (Roche). Bound probes were detected using 3,3′-diaminiobenzidine (DAB) as a substrate (Vector Laboratories). An *in situ* BrdU-uptake assay was used as a surrogate measure of cell proliferation. Pregnant mice were injected intraperitoneally with a solution containing bromodeoxyuridine (BrdU, Sigma B-5002) at a dose of 10 micrograms/g body weight. Mice were sacrificed at two hours post-injection. Kidneys were fixed with 4% PFA overnight at 4°C, dehydrated and embedded in paraffin. BrdU incorporation was detected in 5 micrometer sections using anti-BrdU horseradish peroxidase (1∶50, Roche, USA).

### Transient Transfection of Metanephric Mesenchymal Cells

Metanephric mesenchyme cells were isolated at E11.5 and cultured in Dulbecco’s modified Eagle’s medium (DMEM, GIBCO) supplemented with 15% fetal bovine serum, 100 U/ml penicillin and 100 micrograms/ml streptomycin [45]. Isolated cells were cultured overnight then passaged to a new dish and cultured for 16 hours after which cells were transfected with a pEGFP-N1 vector containing full length *Kif3a* cDNA (0.8 micrograms/well) using Lipofectamine™ 2000 (Invitrogen) according to the manufacturers’ instructions. Cells were harvested 36 hours after transfection.

### Antibodies

Immunofluorescence analyses of kidney tissue and cultured cells was performed using published methods [44], [46], [47] using antibodies directed against: KIF3A (1∶100, K3513, Sigma), PAX2 (1∶500, PRB-276P, Covance), α-AcT (1∶1000, T6793, Sigma), Cytokeratin (1∶200, C2562, Sigma), NCAM (Neural Cell Adhesion Molecule) (1∶50, C9672, Sigma), WT1 (1∶500, sc-192, SANTA CRUZ), LTL (Lotus Tetragonolobus Lectin) (1∶100, FITC, FL-1321, Vector Laboratories, Inc.), SIX2 (1∶200, ab68908, Abcam), Phospho-Histone H3 (Ser28) (1∶200, Cell Signaling) and GFP (1∶1000, Ab16901, Millipore). Secondary antibodies used were Alexa 488 anti-rabbit IgG, anti-rat IgG and anti-chicken IgG, as well as Alexa 546 anti-rat IgG and anti-rabbit IgG, (1∶1000, Molecular Probes, Invitrogen Detection Technologies). DAPI (1∶1000, D9564, Sigma) was used for nuclear staining.

### RNA Isolation and Real-Time PCR

Total RNA was purified from isolated E11.5 ureteric bud and metanephric mesenchyme or whole embryonic kidneys using the RNeasy Mini Kit (QIAGEN). cDNA was synthesized using a first strand cDNA synthesis kit (Invitrogen). Real-time RT-PCR [46] was performed to determine the expression of *Kif3a, Ptc1*, *Gli1 and Fgf8*. *Gapdh* served as an endogenous control. The primers used, the fragments amplified, and the annealing temperatures are detailed in Table S2 in File S1. Quantitative RT-PCR (qRT-PCR) was performed using an Applied Biosystems 7900 HT fast RT-PCR system with TaqMan® Universal PCR Master Mix and TaqMan® probes for *Kif3a* (Mm00492876_m1) or *Fgf8* (Mm00438922_m1). Mouse *Gapdh* was used as an endogenous control (Mm03302249_g1, Applied Biosystems). Primers for *Ptc1* and *Gli1* were designed using Primer 3 software. Relative mRNA expression was determined using the standard curve method. Samples were analysed in triplicates.

### Image Capture and Data Analysis

Kidneys for SEM were perfused with 4% PFA and 2% glutaraldehyde in PBS, prepared as described previously [14], and visualized with a FEI XL30 Scanning Electron Microscope at the Advanced Bioimaging Center of Mount Sinai Hospital, University of Toronto. Microscopy was also performed using a spinning disk confocal laser scanning microscope or Zeiss Axiovision4 light microscope. A minimum of four mice (derived from different litters) were analysed for each developmental stage, gene, antigen and genotype. Student’s t-test (two-tailed) was used to analyze the mean differences between groups. The statistical significance was taken at a value of P<0.05. Images were combined using Adobe Photoshop CS2 and CorelDRAW 14 software.

### Ethics Statement

All experiments using animals have been conducted according to the guidelines adopted by the Toronto Centre for Phenogenomics and which are in accord with national and international guidelines. The experiments, the results of which are reported here, were approved by the Institutional Animal Care and Use Committee (IACUC) of the Toronto Centre for Phenogenomics. Animals were sacrificed via inhalation of CO2.

## Supporting Information

Figure S1 **Primary cilia are present in both ureteric epithelial and metanephric mesenchyme cells in the developing murine kidney.** (A–D) Schematic of ureteric bud epithelial cells (black), metanephric mesenchyme cells and metanephric-derived nephrogenic stuctures during progressive stages of kidney development (green). (A’–D’’) Primary cilia (acetylated α-tubulin, red, arrows) are present in both ureteric (arrows), metanephric mesenchyme cells and their derivatives (Pax2, green, arrow heads) in E11.5 (A’), E13.5 (B’) and E15.5 (C’) kidneys. (A’’–D’’) Single color shows primary cilia in the developing kidney. CM, Condensate Mesenchyme; CSB, Comma-Shape Body; UB, Ureteric Bud; RV, Renal vesical; WT, wild type. Scale bar: C’–F’’, 25 micrometer.
(TIF)

Figure S2 **Expression of** ***Kif3a*** **in kidney tissue.** Ureteric bud was dissected free of metanephric mesenchyme in E11.5 kidney tissue of WT, *Kif3a^−/−UB^*, and *Kif3a^−/−MM^* mice. *Kif3a* mRNA expression was analyzed by quantitative RT-PCR and quantified. *Kif3a* is not expressed in the ureteric bud of *Kif3a^−/−UB^* mice but is expressed in metanephric mesenchyme. *Kif3a* is not expressed in the metanephric mesenchyme of *Kif3a^−/−MM^* mice but is expressed in ureteric bud. (***, P<0.001).
(TIF)

Figure S3 **Decreased nephron number in both** ***Kif3a^−/−UB^*** **and** ***Kif3a^−/−MM^*** **kidneys.** (A–F) Histological sections, stained with hematoxylin and eosin demonstrate a qualitative decrease in the number of glomeruli (arrows) at E15.5 (A,B,C) and E18.5 (D,E,F) in both *Kif3a^−/−UB^* (B, E) and *Kif3a^−/−MM^* (C, F) mice compared to WT (A, D). (G–I) The decrease in mature glomeruli (arrows) in both *Kif3a^−/−UB^* (H) and *Kif3a^−/−MM^* (I) mice is greater at P0. Cysts are present in collecting duct (H, arrowheads) and tubules (I, arrowheads) in both mutant mouse strains. (J) Quantification of the number of mature glomeruli demonstrates a decrease in *Kif3a^−/−UB^* and *Kif3a^−/−MM^* mice at E15.5, E18.5 and P0 compared to controls. (***, P<0.001; **, P<0.01; *, P<0.05). Scale bars: 50 micrometer.
(TIF)

Figure S4 **Cell proliferation and SIX2-positive nephrogenic progenitor cells** **in** ***Kif3a^−/−MM^*** **kidney tissue.** (A, B) *In situ* BrdU incorporation assay in E13.5 kidney tissue. Ureteric bud tip is demarcated by the yellow dotted line. (C) Quantification of BrdU-positive cap mesenchyme cells reveals no significant difference between *Kif3a^−/−MM^* and WT mice. (D, D’) SIX2-positive cells (nephrogenic precursors) are organized in a tightly packed layer around the ureteric bud tip at E13.5 in WT mice. (E, E’) The SIX2-positive cells are disorganized surrounding the ureteric tip in *Kif3a^−/−MM^* mice. (F) Quantification of the SIX2-positive cells demonstrates a significant decrease in *Kif3a^−/−MM^* mice versus WT control mice. (**, P<0.01). Scale bars: 50 micrometer.
(TIF)

Figure S5 **Expression of** ***Fgf8, Wnt4, Lim1*** **and** ***Wnt9b mRNAs*** **in E13.5** ***Kif3a^−/−MM^*** **mice.** Expression was determined by *in situ* hybridization. Expression of *Fgf8* is mildly decreased in *Kif3a^−/−MM^* mice (A’) compared to WT (A) but expression of *Wnt4*. (B, B’), *Lim1* (C, C’) and *Wnt9b* (D, D’) is unchanged. Scale bars: 50 micrometer.
(TIF)

File S1 Table S1, Primers used to genotype the various mutant mouse lines. Table S2, The primers and their RT-PCR products used to estimate the mRNA expression in kidneys and in MM cells.
(DOC)
