# Supplementary figures and images for: Kif3a Controls Murine Nephron Number Via GLI3 Repressor, Cell Survival, and Gene Expression in a Lineage-Specific Manner
Source: PLoS One. 2013 Jun 7;8(6):e65448. doi: 10.1371/journal.pone.0065448 (PMC3676467; doi:10.1371/journal.pone.0065448)

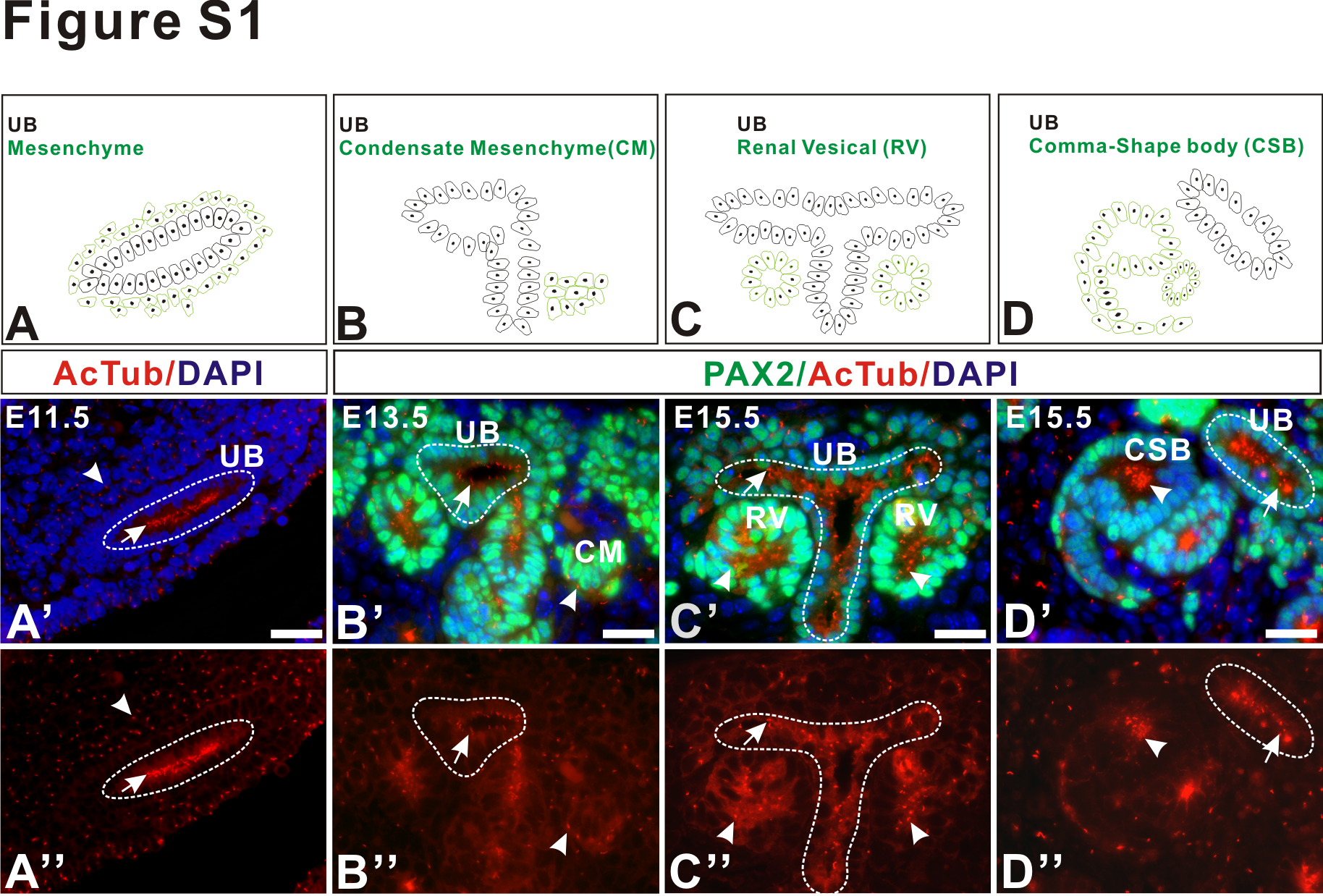

Supplement: Figure S1 — Primary cilia are present in both ureteric epithelial and metanephric mesenchyme cells in the developing murine kidney. (A–D) Schematic of ureteric bud epithelial cells (black), metanephric mesenchyme cells and metanephric-derived nephrogenic stuctures during progressive stages of kidney development (green). (A’–D’’) Primary cilia (acetylated α-tubulin, red, arrows) are present in both ureteric (arrows), metanephric mesenchyme cells and their derivatives (Pax2, green, arrow heads) in E11.5 (A’), E13.5 (B’) and E15.5 (C’) kidneys. (A’’–D’’) Single color shows primary cilia in the developing kidney. CM, Condensate Mesenchyme; CSB, Comma-Shape Body; UB, Ureteric Bud; RV, Renal vesical; WT, wild type. Scale bar: C’–F’’, 25 micrometer. (TIF) [file pone.0065448.s001.tif]

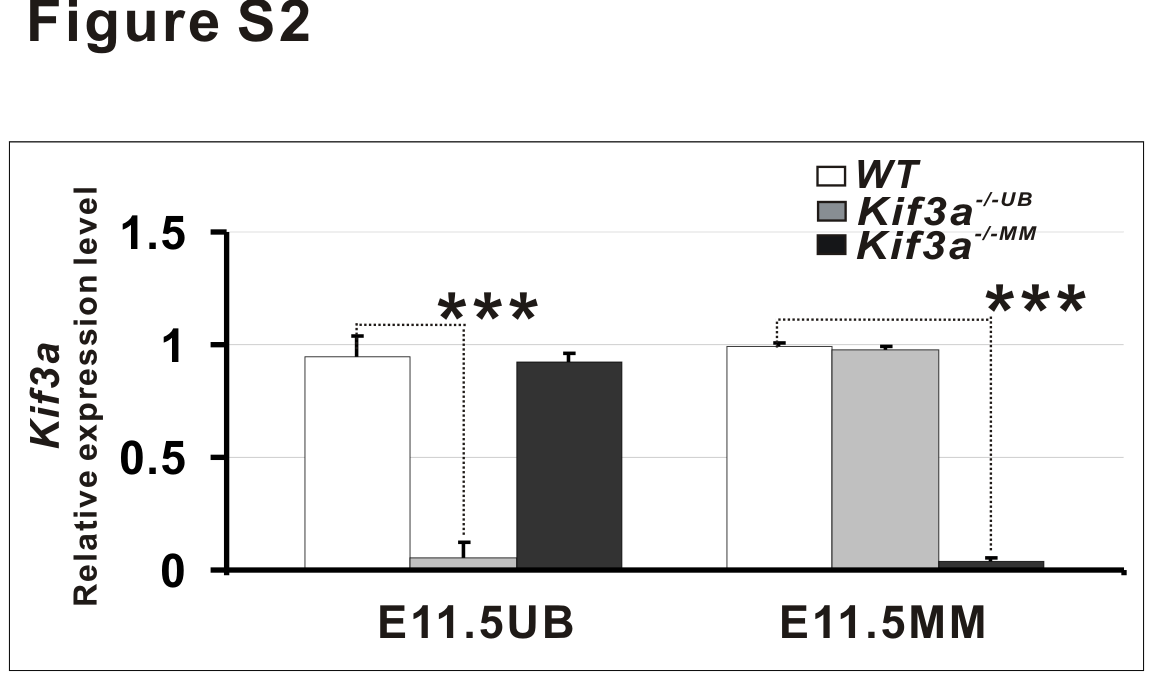

Supplement: Figure S2 — Expression of Kif3a in kidney tissue. Ureteric bud was dissected free of metanephric mesenchyme in E11.5 kidney tissue of WT, Kif3a−/−UB, and Kif3a−/−MM mice. Kif3a mRNA expression was analyzed by quantitative RT-PCR and quantified. Kif3a is not expressed in the ureteric bud of Kif3a−/−UB mice but is expressed in metanephric mesenchyme. Kif3a is not expressed in the metanephric mesenchyme of Kif3a−/−MM mice but is expressed in ureteric bud. (***, P<0.001). (TIF) [file pone.0065448.s002.tif]

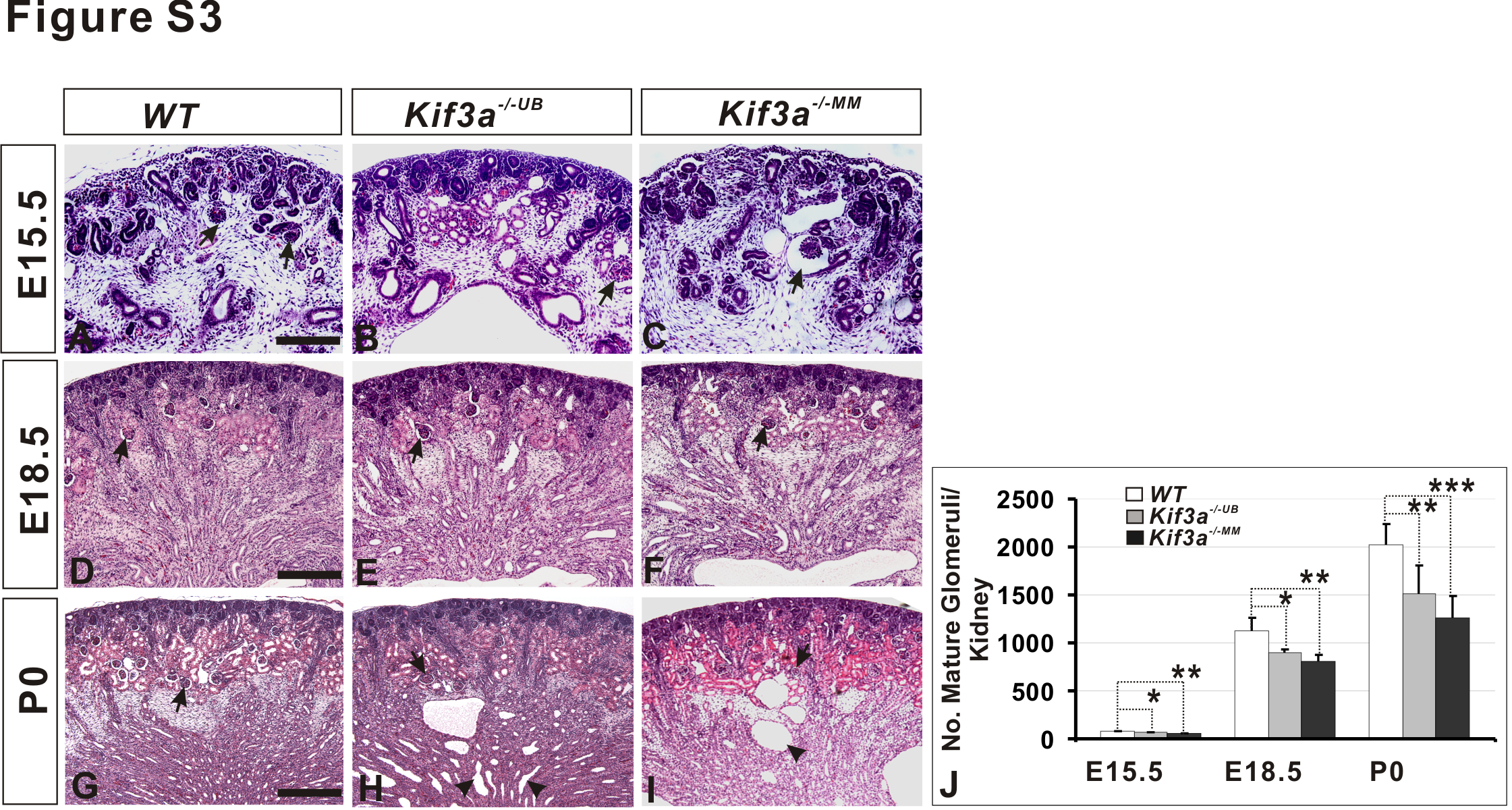

Supplement: Figure S3 — Decreased nephron number in both Kif3a−/−UB and Kif3a−/−MM kidneys. (A–F) Histological sections, stained with hematoxylin and eosin demonstrate a qualitative decrease in the number of glomeruli (arrows) at E15.5 (A,B,C) and E18.5 (D,E,F) in both Kif3a−/−UB (B, E) and Kif3a−/−MM (C, F) mice compared to WT (A, D). (G–I) The decrease in mature glomeruli (arrows) in both Kif3a−/−UB (H) and Kif3a−/−MM (I) mice is greater at P0. Cysts are present in collecting duct (H, arrowheads) and tubules (I, arrowheads) in both mutant mouse strains. (J) Quantification of the number of mature glomeruli demonstrates a decrease in Kif3a−/−UB and Kif3a−/−MM mice at E15.5, E18.5 and P0 compared to controls. (***, P<0.001; **, P<0.01; *, P<0.05). Scale bars: 50 micrometer. (TIF) [file pone.0065448.s003.tif]

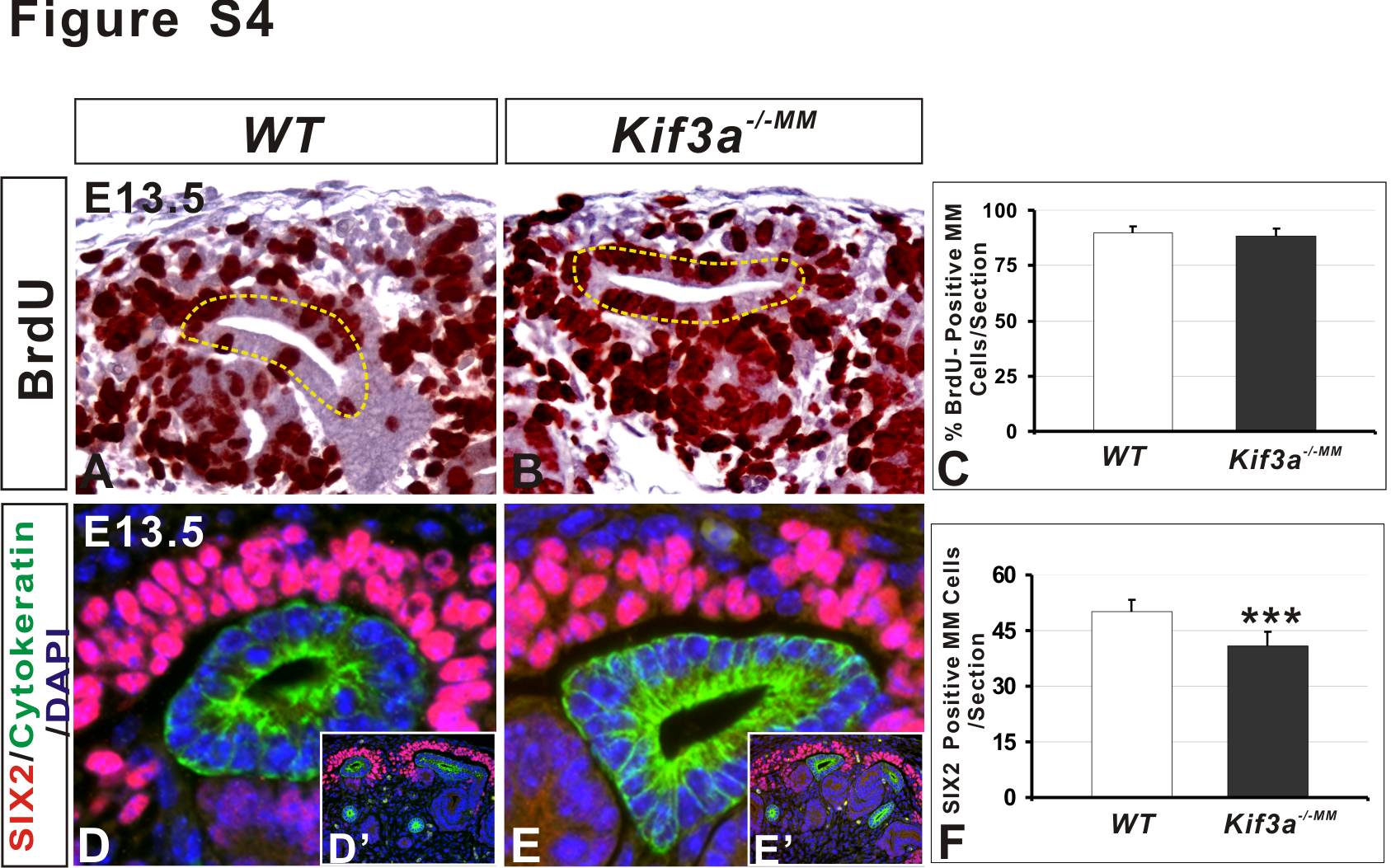

Supplement: Figure S4 — Cell proliferation and SIX2-positive nephrogenic progenitor cells in Kif3a−/−MM kidney tissue. (A, B) In situ BrdU incorporation assay in E13.5 kidney tissue. Ureteric bud tip is demarcated by the yellow dotted line. (C) Quantification of BrdU-positive cap mesenchyme cells reveals no significant difference between Kif3a−/−MM and WT mice. (D, D’) SIX2-positive cells (nephrogenic precursors) are organized in a tightly packed layer around the ureteric bud tip at E13.5 in WT mice. (E, E’) The SIX2-positive cells are disorganized surrounding the ureteric tip in Kif3a−/−MM mice. (F) Quantification of the SIX2-positive cells demonstrates a significant decrease in Kif3a−/−MM mice versus WT control mice. (**, P<0.01). Scale bars: 50 micrometer. (TIF) [file pone.0065448.s004.tif]

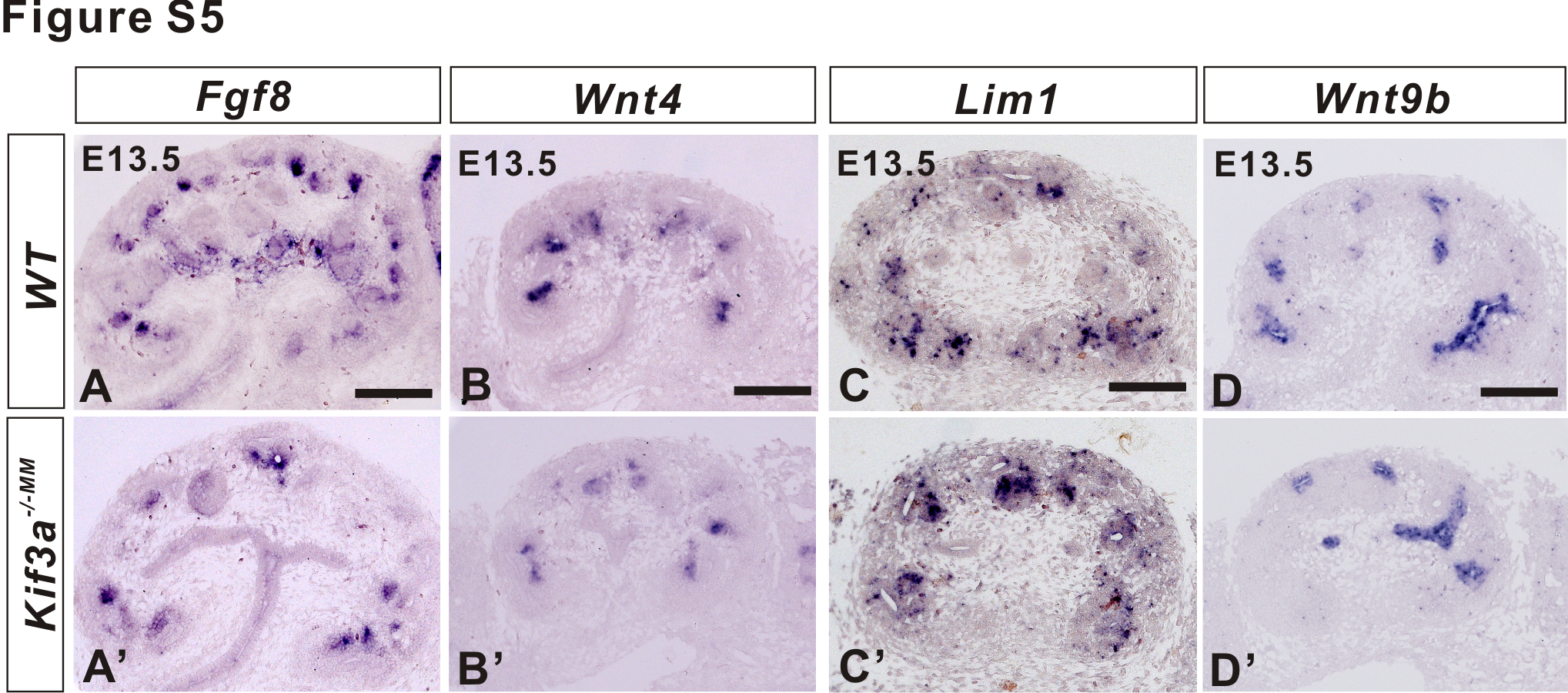

Supplement: Figure S5 — Expression of Fgf8, Wnt4, Lim1 and Wnt9b mRNAs in E13.5 Kif3a−/−MM mice. Expression was determined by in situ hybridization. Expression of Fgf8 is mildly decreased in Kif3a−/−MM mice (A’) compared to WT (A) but expression of Wnt4. (B, B’), Lim1 (C, C’) and Wnt9b (D, D’) is unchanged. Scale bars: 50 micrometer. (TIF) [file pone.0065448.s005.tif]
