# Supplementary material for: Kif3a Controls Murine Nephron Number Via GLI3 Repressor, Cell Survival, and Gene Expression in a Lineage-Specific Manner
Source: PLoS One. 2013 Jun 7;8(6):e65448. doi: 10.1371/journal.pone.0065448 (PMC3676467; doi:10.1371/journal.pone.0065448)
Supplement: File S1 — Table S1, Primers used to genotype the various mutant mouse lines. Table S2, The primers and their RT-PCR products used to estimate the mRNA expression in kidneys and in MM cells. (DOC) [file pone.0065448.s006.doc]

**Supporting Information**

***Kif3a* Controls Murine Nephron Number Via GLI3 Repressor, Cell Survival and Gene Expression in a Lineage-Specific Manner**

Lijun Chi^1,2^, Alevtina Galtseva^1^, Lin Chen^1^, Rong Mo^1^, Chi-chung Hui^1,3^ and Norman D Rosenblum^1,2,4,5,6*^

**Inventory of Supporting Information**

1. Figures S1 to S5

2. Table S1 and S2

3. References for Supporting information

**Table. S1 and S2. Primers used for genotyping**

**Table S1. Primers used to genotype the various mutant mouse lines**

Mouse lines Primers 5’-3’ Fragment (bp) Reference

*Kif3a* 5’- AGGGCAGACGGAAGGGTGG -3’ Flox: 500

5’- TCTGTGAGTTTGTGACCAGCC -3’ WT: 377

5’- TGGCAGGTCATTGGACGCAG-3’ Null: 210 (Marszalek et al., 1999)

*Hoxb7-CreEGFP* 5’- CGCCGCATAACCAGTGAAAC -3’ (Yu et al., 2002)

*Rarb2-Cre*  5’- ATGTCCAATTTACTGACCG -3’ 240 (Kobayashi et al., 2005)

Cre-ER^TM^  (Hayashi et al., 2002)

*R26R*: 5’- AAAGTCGCTCTGAGTTGTTAT-3’

5’-GGA GCGGGAGAAATGGATATG-3’ WT: 550

5’- GCGAAGAGTTTGTCCTCAACC-3’ TG: 300 (Jackson Laboratory, strains: 003474)

**Table S2. The primers and their RT-PCR products used to estimate the mRNA expression in kidneys and in MM cells**

Gene Primers 5’-3’ Annealing temperature Fragment (bp)

*Kif3a* 5’- CGGAAAGCTGCGATAATGTGAAG-3’

5’- GCCTTCCAGAACAGAATCAATAATTGG-3’ 56°C 248

*Fgf8* 5’- GCGAAGGAGAGAAGACGATG-3’

5’- ACGCACATACTAGCGCACAC-3’ 59°C 207

*Ptc1* 5’*-* CTTCCCAAATCTCCCTCCTC-3’

5’- GTGGTTGTTCCTCCGTCAGT-3’ 59°C 108

*Gli1* 5’-AGCCCAGCTCTAGTCCTTCC-3’

5’-GGAGTCCTTGCTCTGTCCTG-3’ 59°C 112

*Gapdh* 5’- TGATGACATCAAGAAGGTGGTGAAG -3’

5’- TCCTTGGAGGCCATGTAGGCCAT -3’ 58°C 248

PCR was initiated in the annealing temperatures that was appropriate to the primer pairs selected. The annealing was followed by 40 cycles of PCR, 10 seconds denaturation at 94°C, 30 seconds annealing at 56°C,58°Cor 59°C depending of the primers used followed by one minute extension reaction at 72°C.

**References:**

[Hayashi S](http://www.ncbi.nlm.nih.gov/pubmed?term=Hayashi S%5BAuthor%5D&cauthor=true&cauthor_uid=11944939) and [McMahon AP](http://www.ncbi.nlm.nih.gov/pubmed?term=McMahon AP%5BAuthor%5D&cauthor=true&cauthor_uid=11944939). (2002) Efficient recombination in diverse tissues by a tamoxifen-inducible form of Cre: a tool for temporally regulated gene activation/inactivation in the mouse**.** [Dev Biol.](http://www.ncbi.nlm.nih.gov/pubmed/11944939?dopt=Citation#%23) *244(2):*305-18.

Kobayashi, A., Kwan, K.M., Carroll, T.J., McMahon, A.P., Mendelsohn, C.L., and Behringer, R.R. (2005). Distinct and sequential tissue-specific activities of the LIM-class homeobox gene Lim1 for tubular morphogenesis during kidney development. Development *132*, 2809-2823.

Marszalek, J.R., Ruiz-Lozano, P., Roberts, E., Chien, K.R., and Goldstein, L.S. (1999). Situs inversus and embryonic ciliary morphogenesis defects in mouse mutants lacking the KIF3A subunit of kinesin-II. Proc Natl Acad Sci U S A *96*, 5043-5048.

Yu, J., Carroll, T.J., and McMahon, A.P. (2002). Sonic hedgehog regulates proliferation and differentiation of mesenchymal cells in the mouse metanephric kidney. Development *129*, 5301-5312.
